# Evolution of Transient Receptor Potential (TRP) Ion Channels in Antarctic Fishes (Cryonotothenioidea) and Identification of Putative Thermosensors

**DOI:** 10.1093/gbe/evac009

**Published:** 2022-02-02

**Authors:** Julia M York, Harold H Zakon

**Affiliations:** Department of Integrative Biology, University of Texas at Austin, USA

**Keywords:** notothenioids, TRP channels, Antarctica, cold evolution

## Abstract

Animals rely on their sensory systems to inform them of ecologically relevant environmental variation. In the Southern Ocean, the thermal environment has remained between −1.9 and 5 °C for 15 Myr, yet we have no knowledge of how an Antarctic marine organism might sense their thermal habitat as we have yet to discover a thermosensitive ion channel that gates (opens/closes) below 10 °C. Here, we investigate the evolutionary dynamics of transient receptor potential (TRP) channels, which are the primary thermosensors in animals, within cryonotothenioid fishes—the dominant fish fauna of the Southern Ocean. We found cryonotothenioids have a similar complement of TRP channels as other teleosts (∼28 genes). Previous work has shown that thermosensitive gating in a given channel is species specific, and multiple channels act together to sense the thermal environment. Therefore, we combined evidence of changes in selective pressure, gene gain/loss dynamics, and the first sensory ganglion transcriptome in this clade to identify the best candidate TRP channels that might have a functional dynamic range relevant for frigid Antarctic temperatures. We concluded that TRPV1a, TRPA1b, and TRPM4 are the likeliest putative thermosensors, and found evidence of diversifying selection at sites across these proteins. We also put forward hypotheses for molecular mechanisms of other cryonotothenioid adaptations, such as reduced skeletal calcium deposition, sensing oxidative stress, and unusual magnesium homeostasis. By completing a comprehensive and unbiased survey of these genes, we lay the groundwork for functional characterization and answering long-standing thermodynamic questions of thermosensitive gating and protein adaptation to low temperatures.

Significance

Many physiological adaptations have their molecular basis in transient receptor potential (TRP) channels, including adaptations of the sensory system to novel thermal environments, yet no thermosensitive proteins have been discovered that function below 10 °C. We comprehensively analyze the evolutionary dynamics of TRP channels within Antarctic cryonotothenioid fishes and propose candidate channels that might be the mechanism for thermosensation in this frigid environment. As the first exploration of genotypes that might underlie thermosensory adaptation in fishes, we identify multiple channels and dozens of sites for functional characterization and put forward multiple hypotheses on the molecular basis of cold adaptation.

## Introduction

Within living systems, all physiological processes and macromolecular structures are affected by temperature, and therefore it is vital for organisms to sense their thermal environment. Much attention has focused on the great variability and fluctuating nature of thermal environments on Earth and the effects these fluctuations have on living systems at various temporal and spatial scales including microhabitats, seasonality, and climate change (Garcia et al. 2014; Scheffers et al. 2014; Shi et al. 2016; Tang et al. 2016). Organisms also alter their thermal environments. This occurs directly over short timescales, for example by migration, range shifts, or during development, but also indirectly over longer timescales through the capture and release of greenhouse gases (Duarte et al. 2005; Singh et al 2010; Chen et al. 2011; IPCC 2014; Ahlström et al 2015; Cheddadi et al. 2016; Shaw 2016; Truebano et al. 2018; Donelson et al. 2019). The energy exchange between the thermal environment on Earth and the organisms within that environment is thus dynamic and ancient and has required organisms to evolve sensory mechanisms that reliably inform them of ecologically and physiologically relevant thermal variation.

Our knowledge of thermosensory mechanisms has focused on ion channels: integral membrane proteins that open (or gate) in response to various stimuli, transducing environmental changes into cellular electrical signals. Thermal environments vary widely on Earth, between −98 and 1,250 °C, but 90% of the biosphere is at 10 °C or lower (Gianese et al. 2001; Poland et al. 2014; Scambos et al. 2018). Yet we have not discovered a temperature-sensitive channel that gates below 10 °C (Story et al. 2003; Oda et al. 2018; Buijs and McNaughton 2020). We are, therefore, lacking an understanding of how organisms might discriminate between the majority of thermal environments they could encounter. One of the most extreme such environments is the frigid Southern Ocean that surrounds Antarctica—a stable thermal environment that today ranges at most between −1.9 and +4 °C (Picken 1985). Ectotherms in this environment may encounter multiple years where the water does not warm above the freezing point of their body fluids (Cziko et al. 2014). In this study, we identify the best candidates for thermosensory mechanisms in a group of teleost fishes that diversified in the Southern Ocean: the cryonotothenioids (Near et al. 2015). Thermal tolerance studies indicate that cryonotothenioids have some of the lowest critical thermal maxima of teleost fishes—lethality begins at just 6 °C in some species (Somero and DeVries 1967; Bilyk and DeVries 2011).

**Fig. 1.**
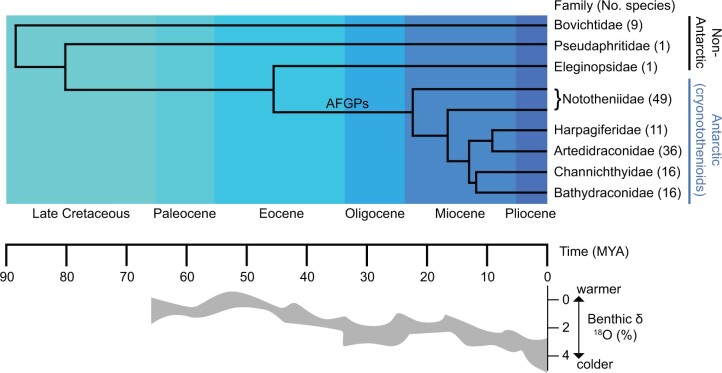
Estimated divergence times and phylogenetic relationships between the families of the notothenioidei suborder (notothenioids), with oxygen isotope composition (δ^18^O) of benthic foraminifera, which is a proxy for temperature and also ice volume (Shevenell et al. 2004). Gray-shaded area indicates the range in δ^18^O values measured. Branch where antifreeze glycoproteins (AFGPs) arose is indicated. Note that Nototheniidae is paraphyletic. Colored boxes indicate epochs. Phylogeny and isotope data adapted from Near et al. (2012) and Near et al. (2015); species numbers from Eastman and Eakin (2021).

The Notothenioidei suborder is an ideal study system for the evolution of thermosensation because within this clade five families (the cryonotothenioids) are found primarily in the Antarctic while the three more basal families are non-Antarctic (fig. 1; Near et al. 2015). This provides an opportunity for comparison of the temperate, non-Antarctic clades with the Antarctic cryonotothenioid clade, which has diversified in an extreme and specific thermal environment. The common ancestor of cryonotothenioids split from the more basal non-Antarctic clades during a period of global cooling and Antarctic glaciation, evolved antifreeze glycoproteins, and diversified into the currently recognized 128 species in one of the fastest rates of speciation in marine teleosts (Chen et al. 1997; Near et al. 2012; Rabosky et al. 2018; Eastman and Eakin 2021). It is estimated that Southern Ocean surface temperatures have not exceeded 5 °C in the last 15 Myr, and the major pulses of diversification have primarily occurred within that time, beginning in the late Miocene (Pörtner 2006; Near et al. 2012). High latitude Antarctic species today rarely encounter temperatures above +1 °C (Hunt et al. 2003; DeVries and Cheng 2005; Cziko et al. 2014; Arana et al. 2020). Meanwhile, the non-Antarctic clades are composed of relatively few species, such as *Cottoperca gobio* (Bovichtidae) and *Eleginops maclovinus* (Eleginopsidae), and are considered eurythermal—experiencing thermal environments typically ranging from 4 to 10 °C (but might encounter 0–15 °C extremes; Verde et al. 2012; Oyarzún-Salazar et al. 2021).

The best-studied molecular mechanism underlying thermosensation in metazoans are transient receptor potential (TRP) channels (Hoeffstaetter et al. 2018). TRP channels are ancient: orthologs are present in fungi, protists, and chlorophyte algae, indicating they evolved in a single-celled eukaryotic ancestor more than 1.5 billion years ago (Cai and Clapham 2012; McGoldrick et al. 2019). TRP channels are typically nonspecific cation channels composed of four subunits; each subunit has at least six transmembrane domains (S1–S6), and the S5 and S6 domains of each subunit form the pore where ions are conducted through the membrane (Gees et al. 2012). The cytosolic N- and C-terminal domains vary widely in sequence and structure while the transmembrane and extracellular domains are more conserved. Within the TRP channel superfamily, ten subfamilies have been identified based on sequence (TRPA, TRPC, TRPM, TRPML, TRPN, TRPP, TRPS, TRPV, TRPVL, and TRPY/TRPF; Martinac et al. 2008; Peng et al. 2015; Himmel et al. 2021). Of these, seven are found in metazoans, and temperature-sensitive TRP channels (thermoTRPs) have been identified in the TRPA, TRPC, TRPM, TRPP, and TRPV subfamilies (Patapoutian et al. 2003; Gallio et al. 2011; Castillo et al. 2018). TRP channels are polymodal: depending on the channel they might be activated by heat, ligands, light, pH, pressure, voltage, or a combination thereof. At least one thermoTRP has been characterized in algae, indicating that thermosensitive gating may be an ancestral property of TRP channels (McGoldrick et al. 2019).

All cellular processes and structures are thermosensitive to some extent, but thermoTRPs show a >5-fold change in activity over a 10 °C temperature range (*Q*_10_ < 0.2 or *Q*_10_ > 5; Voets 2012). Each thermoTRP has a specific dynamic range of temperatures within which it functions and some thermoTRPs show distinct activation thresholds while others activate more gradually. Although most TRP channels are activated by an increase in temperature, a few are activated by cooling. ThermoTRPs are highly expressed in the sensory nerves such as the trigeminal and dorsal root ganglia, but are often also expressed in nonexcitable tissues where it is expected that they do not have a direct thermosensitive function (Nilius and Owsianik 2011; Gees et al. 2012). In the sensory neurons, the channels gate in response to a temperature change and alter the membrane voltage sending a signal to the brain. Downstream neurons mediate appropriate behavioral responses, such as escape (Haesemeyer et al. 2018).

Although the majority of discoveries on the thermosensitive function of TRP channels are from work on mammals and *Drosophila*, these functions appear to be conserved in fish (Saito and Tominaga 2017; Castillo et al. 2018). Most of the mammalian thermoTRP genes are conserved in teleosts (Saito and Tominaga 2015). A few channels from fish are temperature-gated in vitro including TRPV1 from zebrafish and TRPA1 paralogs from zebrafish, medaka, and pufferfish (Gau et al. 2013; Oda et al. 2016, 2017, 2018). In vivo work on zebrafish indicates that knockdowns of TRPV1 blunt behavioral responses to noxious temperature stimuli (Gau et al. 2013). Little else is known about thermoTRPs in fishes.

In this study, we assume notothenioid fishes can sense temperature and do so primarily with the peripheral, and not the central, nervous system. We caveat that some teleosts are known to have heat-sensitive neurons in the brain, and further, some aquatic Antarctic invertebrates show no escape responses from lethally high temperatures (Boulant and Dean 1986; Bates et al. 2010). However, evidence suggests Antarctic notothenioids do sense and escape noxious thermal environments—although data are limited (Crawshaw and Hammel 1971; Macdonald et al. 1987; Fanta et al. 1989; Robinson 2008). In one example, non-Antarctic *E. maclovinus*, as well as the secondarily temperate *Harpagifer bispinis*, which diverged from its Antarctic sister *H. antarcticus* 1.7–0.8 Ma, demonstrated robust temperature preferences that depended strongly on acclimation temperature (Hüne et al. 2015; Lattuca et al. 2018; Giménez et al. 2021).

It is likely cryonotothenioids retain some ability to sense thermal stimuli as it is critical for them to avoid even relatively moderate increases in temperature. *Harpagiferantarcticus* had an acute inflammatory response after being held at 6 °C for just 48 h, and temperatures >8 °C are lethal (Thorne et al. 2010; Navarro et al. 2019). Intrusions of warm circumpolar deep water can rapidly warm waters in the Antarctic shelf by nearly 3 °C (Isla and Gerdes 2019). Some parts of the Southern Ocean have warmed an average of 1 °C in the last 50 years and are predicted to warm 0.1 °C per decade on average in the future. This warming is regionally variable, however, and some areas of the Antarctic shelf are predicted to cool (Meredith and King 2005; Schmidtko et al. 2014). Thus, the future existence of these specialist fish may rely on their ability to discriminate temperatures.

To identify the best candidate TRP channels that could function as thermosensors in cryonotothenioid fishes we 1) mined existing transcriptomic data to identify coding sequences across the notothenioid clade, 2) tested coding sequences for evidence of changes in selective pressure relative to other species, 3) examined notothenioid genomes for TRP channel gene duplication and loss, 4) generated the first sensory ganglion-specific transcriptome for notothenioid fishes, and 5) selected candidate genes for site-specific investigation of diversifying selection. We hypothesize that a small subset of cryonotothenioid TRP channels will be highly expressed in the sensory ganglia and show evidence of intensified or diversified selective pressure in cryonotothenioids, both across the gene tree and the primary protein sequence.

## Results

### Existing Transcriptomic Data

**Fig. 2.**
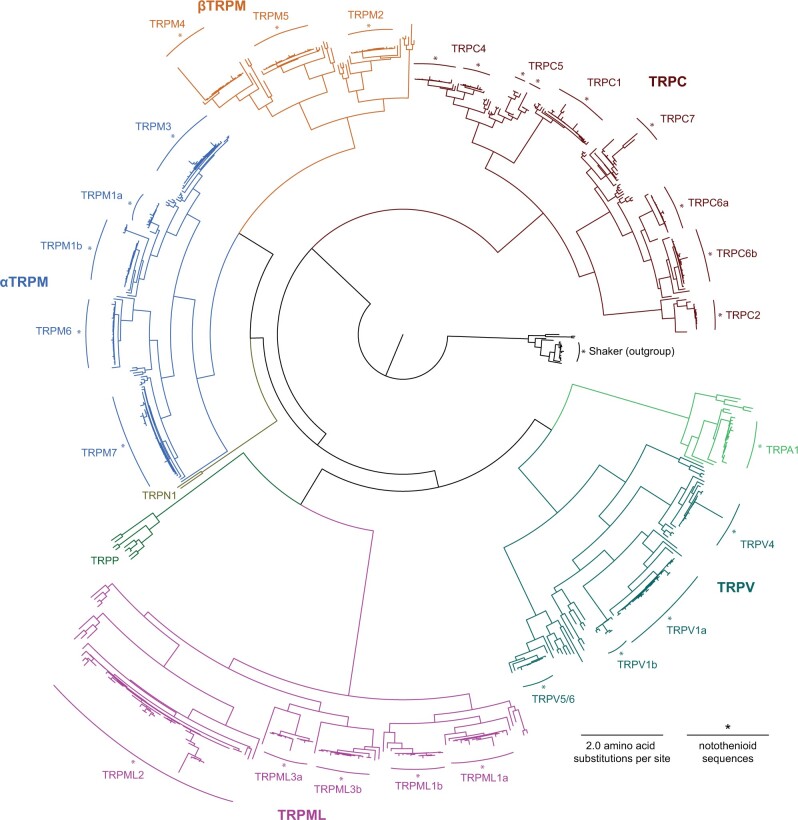
Maximum likelihood phylogenetic relationships of all predicted protein sequences from existing transcriptomic notothenioid data and reference TRP sequences. Colors indicate TRP channel subfamilies. Notothenioids clusters indicated by asterisks (see Supplementary Material online for tip labels, species, data sources, and sequence accessions). No notothenioid proteins clustered with TRPP or TRPN subfamilies, but all other subfamilies were represented in notothenioid transcriptomic data.

We mined 13 existing notothenioid transcriptomes from 12 species representing five families: Eleginopsidae, Nototheniidae, Harpagiferidae, Bathydraconidae, and Channichthyidae (see supplementary table S1, Supplementary Material online for species, tissues, and sources). When multiple tissues were sampled, sequences from all tissues were pooled. We found notothenioids expressed transcriptomic sequences that clustered with most of the known vertebrate TRP channels in a maximum likelihood phylogeny. The full tree generated with all the predicted protein sequences from notothenioid transcriptomes is in figure 2 (for tip labels and bootstrap support values, see supplementary fig. S1, Supplementary Material online).

TRPM1, TRPV1, TRPC4, TRPML1, TRPC6, and TRPML3 proteins clustered as “a” and “b” clades, which appear to be due to the ancestral teleost genome duplication (supplementary figs. S1–S3, Supplementary Material online). All teleosts lack *TRPM8*, *TRPV2*, and *TRPV3*, and no notothenioid protein sequences clustered with reference sequences for these channels (Saito and Tominaga 2015). A single cluster of notothenioid proteins grouped with both TRPV5 and TRPV6 reference sequences and thus was annotated TRPV5/6. No TRPC3, TRPP, or TRPN proteins were found in the existing notothenioid transcriptome data. A few notothenioid sequences resulted in long branches and these sequences were checked for the presence of other genes. We otherwise found no reason to trim or exclude them. Accessions for all sequences can be found in supplementary table S2, Supplementary Material online.

### Changes in Selection Pressure

We used RELAX from the HyPhy package to test each TRP channel identified in the transcriptome analysis for evidence of unchanged, intensified, or relaxed selection in the cryonotothenioid clade relative to non-Antarctic *E. maclovinus* and the non-notothenioid reference species (Wertheim et al. 2015).

**Fig. 3.**
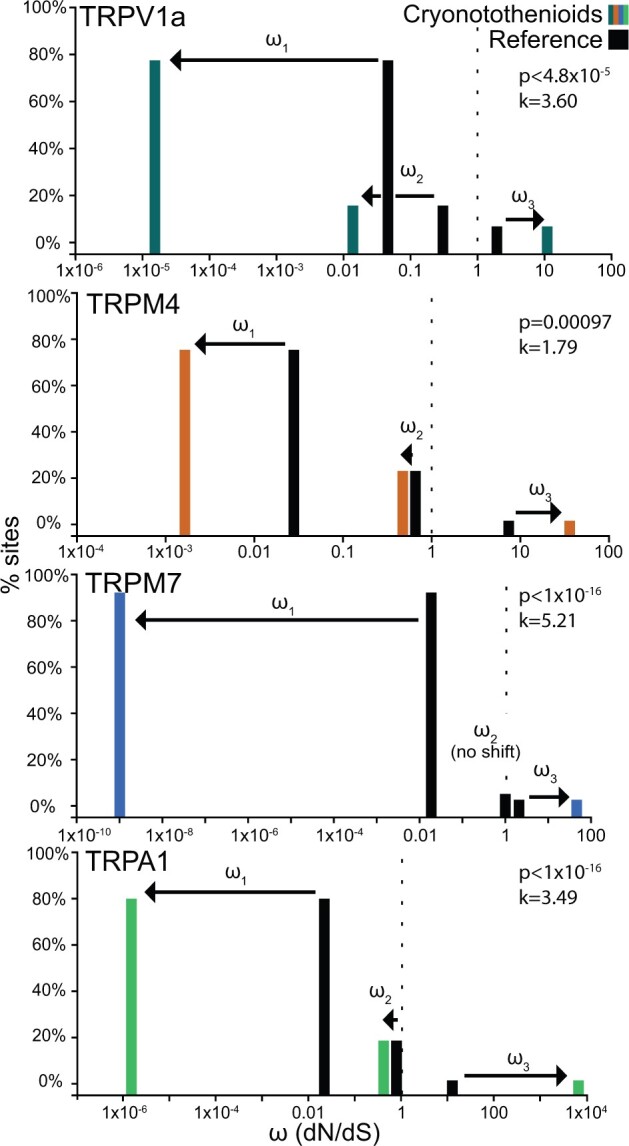
Omega (*ω*) shifts for *TRPV1a*, *TRPM4*, *TRPM7*, and *TRPA1b* channels comparing cryonotothenioid branches (colors) with all other reference branches (black) generated with RELAX (see Materials and Methods). RELAX classifies codons into three classes (*ω*_1_, *ω*_2_, and *ω*_3_) based on nonsynonymous/synonymous substitution rates (d*N*/d*S* or *ω*) and then compares the shifts in distribution of these classes (arrows) in test branches compared with reference branches. All four of these genes showed evidence of significantly intensified selection (*k* values > 1; see table 1 for more details). Colors indicate gene subfamilies, as in figure 2.

We analyzed each channel coding sequence three times, parsing the input data in different ways to probe the clarity of the selection signal. We found that *TRPA1b*, *TRPC4a*, *TRPM4*, *TRPM7*, and *TRPV1a* showed evidence of significant intensification of selection in the cryonotothenioids compared with *E. maclovinus* and reference species (see fig. 3 and table 1 for statistics). In this case, intensification of selection indicates either increased purifying and/or positive selection, as sites are expected to be under different selective pressures in different areas of the protein. *TRPC2*, *TRPC4b*, *TRPC5*, *TRPC7*, *TRPM1b*, *TRPM2*, *TRPM3*, *TRPML1a*, *TRPML3b*, *TRPV1b*, and *TRPV4* all showed evidence for relaxation of selection. *TRPC1*, *TRPC6b*, *TRPM1a*, *TRPML2*, and *TRPML3a* were found to have significant but mixed results, depending on the input data. *TRPC6a*, *TRPM5*, *TRPM6*, and *TRPV5/6* showed no indication of a change in selective pressure in the cryonotothenioids compared with the reference sequences.

**Table 1:** Results of Tests for Selection Intensification or Relaxation Using RELAX from the HyPhy Package

Gene	Data Set	Gblocks Parameters^a^	Result^b^	*P* Value	*K* ^c^	LRT^d^	d*N*/d*S* Test^e^	d*N*/d*S* Reference
*TRPA1b*	All data^f^	Less stringent	**Intensification**	**<1.00E-16**	**3.49**	**130.95**	0.267	0.133
*TRPA1b*	Selected^g^	Less stringent	**Intensification**	**5.05E-05**	**2.88**	**16.43**	0.199	0.154
*TRPA1b*	Selected	More stringent	Neither	0.925174	1.03	0.0088	0.235	0.100
*TRPC1*	All data	Less stringent	**Relaxation**	**0.002722**	**0.82**	**8.98**	0.361	0.031
*TRPC1*	Selected	Less stringent	**Intensification**	**3.37E-10**	**13.07**	**39.45**	0.208	0.034
*TRPC1*	Selected	More stringent	Neither	0.535717	0.95	0.38	0.217	0.024
*TRPC2*	All data	Less stringent	**Relaxation**	**0.012157**	**0.45**	**6.29**	0.177	0.074
*TRPC2*	Selected	Less stringent	Neither	0.472332	0.84	0.52	0.197	0.077
*TRPC2*	Selected	More stringent	**Relaxation**	**0.029482**	**0.57**	**4.74**	0.125	0.066
*TRPC4a*	All data	Less stringent	**Intensification**	**0.004061**	**3.04**	**8.26**	0.238	0.047
*TRPC4a*	Selected	Less stringent	Neither	0.635377	0.95	0.22	0.183	0.073
*TRPC4a*	Selected	More stringent	Neither	0.958547	0.99	0.0027	0.126	0.040
*TRPC4b*	All data	Less stringent	Neither	0.908788	1.01	0.013	0.441	0.048
*TRPC4b*	Selected	Less stringent	**Relaxation**	**8.41E-05**	**0.41**	**15.46**	0.329	0.054
*TRPC4b*	Selected	More stringent	**Relaxation**	**5.34E-05**	**0.46**	**16.32**	0.223	0.034
*TRPC5*	All data	Less stringent	**Relaxation**	**0.000526**	**0.31**	**12.02**	0.238	0.024
*TRPC5*	Selected	Less stringent	**Relaxation**	**2.19E-12**	**0.57**	**49.31**	0.232	0.037
*TRPC5*	Selected	More stringent	**Relaxation**	**4.63E-06**	**0.49**	**20.99**	0.318	0.028
*TRPC6a*	All data	Less stringent	Neither	0.099879	0.79	2.71	0.500	0.044
*TRPC6a*	Selected	Less stringent	Neither	0.367186	0.86	0.81	0.180	0.056
*TRPC6a*	Selected	More stringent	Neither	0.092434	0.71	2.83	0.061	0.029
*TRPC6b*	All data	Less stringent	**Relaxation**	**1.85E-05**	**0.32**	**18.34**	0.465	0.081
*TRPC6b*	Selected	Less stringent	**Intensification**	**7.44E-15**	**11.44**	**60.48**	0.441	0.083
*TRPC6b*	Selected	More stringent	**Intensification**	**4.88E-15**	**39.01**	**61.29**	0.448	0.062
*TRPC7*	All data	Less stringent	**Relaxation**	**6.66E-16**	**0.68**	**65.34**	0.404	0.037
*TRPC7*	Selected	Less stringent	Neither	0.225332	0.89	1.47	0.173	0.038
*TRPC7*	Selected	More stringent	**Relaxation**	**0.006558**	**0.62**	**7.39**	0.173	0.033
*TRPM1a*	All data	Less stringent	Neither	0.408878	1.04	0.68	0.106	0.036
*TRPM1a*	Selected	Less stringent	**Relaxation**	**0.01402**	**0.80**	**6.04**	0.116	0.040
*TRPM1a*	Selected	More stringent	**Intensification**	**0.005569**	**50.00**	**7.68**	0.698	0.016
*TRPM1b*	All data	Less stringent	Neither	0.952851	0.99	0.0035	0.140	0.043
*TRPM1b*	Selected	Less stringent	**Relaxation**	**8.76E-06**	**0.51**	**19.76**	0.212	0.045
*TRPM1b*	Selected	More stringent	N/A					
*TRPM2*	All data	Less stringent	Neither	0.270239	0.41	1.22	0.398	0.138
*TRPM2*	Selected	Less stringent	Neither	0.143224	0.70	2.14	0.279	0.146
*TRPM2*	Selected	More stringent	**Relaxation**	**3.85E-02**	**0.45**	**4.28**	0.221	0.118
*TRPM3*	All data	Less stringent	**Relaxation**	**0.023686**	**0.93**	**5.12**	0.370	0.026
*TRPM3*	Selected	Less stringent	**Relaxation**	**0.021499**	**0.79**	**5.29**	0.228	0.049
*TRPM3*	Selected	More stringent	Neither	0.503541	1.07	0.45	0.110	0.018
*TRPM4*	All data	Less stringent	Neither	0.061874	1.50	3.49	0.354	0.141
*TRPM4*	Selected	Less stringent	**Intensification**	**0.000972**	**1.79**	**10.88**	0.345	0.142
*TRPM4*	Selected	More stringent	**Intensification**	**5.30E-05**	**2.25**	**16.34**	0.333	0.124
*TRPM5*	All data	Less stringent	Neither	0.737634	1.02	0.11	0.250	0.073
*TRPM5*	Selected	Less stringent	Neither	**0.648904**	**1.03**	**0.21**	0.218	0.082
*TRPM5*	Selected	More stringent	Neither	0.223132	1.30	1.48	0.100	0.062
*TRPM6*	All data	Less stringent	Neither	0.761275	0.93	0.092	0.441	0.107
*TRPM6*	Selected	Less stringent	Neither	0.116635	0.80	2.46	0.307	0.150
*TRPM6*	Selected	More stringent	Neither	0.303691	0.85	1.06	0.149	0.095
*TRPM7*	All data	Less stringent	**Intensification**	**<1.00E-16**	**5.21**	**119.26**	0.408	0.080
*TRPM7*	Selected	Less stringent	**Intensification**	**<1.00E-16**	**5.85**	**112.60**	0.268	0.083
*TRPM7*	Selected	More stringent	Neither	0.175015	1.29	1.84	0.138	0.051
*TRPML1a*	All data	Less stringent	Neither	0.415754	1.68	0.66	0.367	0.089
*TRPML1a*	Selected	Less stringent	**Relaxation**	**0.021323**	**0.27**	**5.30**	0.284	0.094
*TRPML1a*	Selected	More stringent	**Relaxation**	**0.00041**	**0.39**	**12.49**	0.281	0.063
*TRPML1b*	All data	Less stringent	Neither	0.509727	1.59	0.43	0.275	0.097
*TRPML1b*	Selected	Less stringent	Neither	0.229514	1.39	1.44	0.312	0.098
*TRPML1b*	Selected	More stringent	Neither	0.171709	1.62	1.87	0.236	0.099
*TRPML2*	All data	Less stringent	**Relaxation**	**<1.00E-16**	**0.49**	**142.38**	0.610	0.142
*TRPML2*	Selected	Less stringent	**Relaxation**	**<1.00E-16**	**0.46**	**119.20**	0.534	0.141
*TRPML2*	Selected	More stringent	**Intensification**	**0.007095**	**5.71**	**7.25**	0.420	0.125
*TRPML3a*	All data	Less stringent	**Intensification**	**5.56E-09**	**13.01**	**33.98**	0.523	0.066
*TRPML3a*	Selected	Less stringent	**Relaxation**	**0.000329**	**0.35**	**12.90**	0.349	0.084
*TRPML3a*	Selected	More stringent	**Relaxation**	**4.65E-05**	**0.28**	**16.59**	0.420	0.073
*TRPML3b*	All data	Less stringent	**Relaxation**	**4.90E-10**	**0.05**	**38.72**	0.707	0.116
*TRPML3b*	Selected	Less stringent	**Relaxation**	**1.63E-06**	**0.17**	**22.99**	0.591	0.116
*TRPML3b*	Selected	More stringent	**Relaxation**	**6.89E-06**	**0.21**	**20.22**	0.477	0.090
*TRPV1a*	All data	Less stringent	Neither	0.518847	1.02	0.42	0.709	0.171
*TRPV1a*	Selected	Less stringent	Neither	0.324557	0.83	0.97	0.708	0.171
*TRPV1a*	Selected	More stringent	**Intensification**	**4.93E-05**	**3.60**	**16.47**	0.645	0.144
*TRPV1b*	All data	Less stringent	**Relaxation**	**0.000632**	**0.45**	**11.68**	0.366	0.124
*TRPV1b*	Selected	Less stringent	**Relaxation**	**0.027226**	**0.30**	**4.88**	0.219	0.131
*TRPV1b*	Selected	More stringent	Neither	0.07112	0.48	3.26	0.153	0.114
*TRPV4*	All data	Less stringent	**Relaxation**	**0.006574**	**0.76**	**7.39**	0.218	0.081
*TRPV4*	Selected	Less stringent	Neither	0.840317	1.05	0.041	0.143	0.088
*TRPV4*	Selected	More stringent	Neither	0.762177	1.06	0.092	0.079	0.068
*TRPV5/6*	All data	Less stringent	Neither	0.052645	1.34	3.76	0.222	0.148
*TRPV5/6*	Selected	Less stringent	Neither	0.927805	1.03	0.0082	0.128	0.146
*TRPV5/6*	Selected	More stringent	Neither	0.794004	0.82	0.068	0.208	0.132

a Gblocks parameters describe the stringency of the alignment trimming.

b Bold indicates significant results.

c *K* parameter indicates selection intensification (*k* > 1) or relaxation (*k* < 1) in test branches compared with reference branches.

d LRT is the likelihood ratio test comparing likelihood of the null and alternative models.

e d*N*/d*S* test and reference refer to nonsynonymous/synonymous substitution ratios of the collective test or reference branches, respectively.

f “All data” indicates the data set includes all of the available transcriptomic sequences.

g “Selected” indicates the data set was limited to only the most complete notothenioid sequences.

### Gene Duplications and Losses

The absence of a sequence from transcriptomic data does not necessarily indicate gene loss and similarly the presence of variable transcripts could be the product of alternative splicing rather than gene duplication. Therefore, we analyzed genomes for seven species from six notothenioid families for TRP channel gene counts (see table 2 and supplementary table S3, Supplementary Material online). The genomic data includes both non-Antarctic *C. gobio* (Bovichtidae) and *E. maclovinus* (Eleginopsidae). Cryonotothenioid species used for genomic analysis were *Dissostichus mawsoni*, *Nototheniacoriiceps* (Nototheniidae), *H. antarcticus* (Harpagiferidae), *Parachaenichthys charcoti* (Bathydraconidae), and *Chaenocephalus aceratus* (Channichthyidae). Two families were not represented in the genomic data: non-Antarctic Pseudaphritidae and Antarctic Artedidraconidae.

**Table 2:** TRP Channel Gene Count for All Genomes Analyzed

			Cryonotothenioids				
	Bovichtidae	Eleginopsidae	Nototheniidae		Harpagiferidae	Channichthyidae	
Gene	*Cottoperca gobio*	*Eleginops maclovinus*	*Dissostichus mawsoni*	*Notothenia coriiceps*	*Harpagifer antarcticus*	*Parachaenichthys charcoti*	*Chaenocephalus aceratus*
*TRPA1b*	1	1	2	1	2	1	2
*TRPC1*	1	1	1	1	1	1	1
*TRPC2*	1	1	1	1	1	1	1
*TRPC3*	0	0	0	0	0	0	0
*TRPC4*	1	2	2	2	2	2	2
*TRPC5*	0	1	1	1	Partial	1	0
*TRPC6a*	1	1	2	1	1	1	1
*TRPC6b*	1	1	1	1	1	1	1
*TRPC7*	1	1	1	1	1	1	1
*TRPM1a*	2	0	1	1	1	1	2
*TRPM1b*	0	1	1	1	1	1	1
*TRPM2*	1	1	1	1	1	1	1
*TRPM3*	1	1	1	1	1	1	1
*TRPM4*	1	1	1	1	1	1	1
*TRPM5*	1	1	1	1	1	1	1
*TRPM6*	1	1	1	1	1	1	1
*TRPM7*	1	1	1	1	1	1	1
*TRPM8*	0	0	0	0	0	0	0
*TRPML1a*	1	1	1	1	1	1	1
*TRPML1b*	1	1	1	1	1	1	2
*TRPML2*	1	1	1	1	1	1	1
*TRPML3a*	0	1	1	1	1	1	1
*TRPML3b*	0	1	1	1	2	1	1
*TRPV1a*	1	1	1	1	1	1	1
*TRPV1b*	1	1	1	1	1	1	1
*TRPV4*	1	1	1	1	1	1	1
*TRPV5/6*	1	1	1	1	1	1	1
*TRPN1*	0	0	0	0	0	0	0
*TRPP1*	0	0	0	0	0	0	0
*TRPP2*	1	1	1	1	1	1	1
*TRPP3*	0	0	0	0	0	0	0
*TRPP5*	0	0	0	0	0	0	0
*Shaker*	1	1	1	1	1	1	1
Total	23	26	29	27	29	27	29

*H. antarcticus* had two duplications in the *TRPML* family: a tandem duplication of part of *TRPML2* and a complete duplication of *TRPML3b*. In the case of *TRPML3b*, the genes immediately upstream (*dynein axonemal intermediate chain 3*) and downstream (*G-protein-signalling modulator 2-like*) were also duplicated, indicating a larger scale duplication than *TRPML3b* alone.

We found no evidence of a *TRPC5* gene in *C. aceratus*. To confirm genomic loss of *TRPC5*, we explored conserved synteny around the gene. In species with *TRPC5*, the gene *canopy FGF signaling regulator 3* (*cnpy3*) was immediately downstream. This *cnpy3* gene was also not found in *C. aceratus*, indicating that sequencing in this region might be incomplete. In the stickleback (*Gasterosteus aculeatus*), the *TRPC5* gene is found at the very beginning of a chromosome, and the upstream region is highly repetitive. In the notothenioids, no conserved sequences or synteny could be resolved in this region upstream of *TRPC5*. However, *TRPC5* was also not found in the *C. aceratus* transcriptome, which should not be subject to the same sequencing difficulties, indicating that the gene might be lost.

Both *C. gobio* and *C. aceratus* had independent duplications of *TRPM1a* (supplementary fig. S2, Supplementary Material online). For *C. gobio*, two copies of *TRPM1a* were found in tandem, whereas in *C. aceratus* the two genes were found on separate scaffolds. In *E. maclovinus*, we found neither the *TRPM1a* gene nor any *TRPM1a* syntenic genes, indicating this may be incomplete sequencing in this region for this species.

**Fig. 4.**
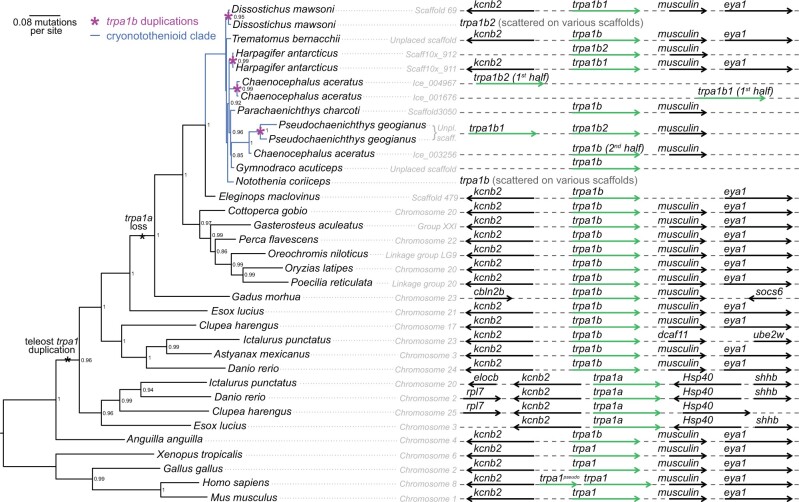
*TRPA1* gene tree with synteny. Gene duplications or losses are indicated by asterisks. Four potential independent duplications of *TRPA1b* (pink asterisks) were found within the cryonotothenioid clade (blue). Some genes were pieced together from several scaffolds (“scattered on various scaffolds”). Node support values are bootstraps. Gray dotted lines indicate connected chromosome or scaffold, arrows indicate gene directionality. *TRPA1* orthologs or paralogs are indicated by green arrows.

The sole vertebrate representative of the TRPA family, *TRPA1*, duplicated in an ancestral teleost into *TRPA1a* and *TRPA1b—*perhaps in the ancestral teleost whole genome duplication (Glasauer and Neuhauss 2014). The *TRPA1a* copy was subsequently lost around evolution of Neoteleostei (and possibly independently in Elopomorpha, as no *TRPA1a* was found in *Anguilla anguilla*). This is indicated by the presence of both genes in species representing Esociformes, Cypriniformes, Clupeiformes, Characiformes, and Siluriformes but only *TRPA1b* in species from Gadiformes, Cyprinodontiformes, Beloniformes, Cichliformes, and Perciformes (fig. 4; Hughes et al. 2018). The lack of *TRPA1a* in the notothenioid genomes was confirmed by examining the regions around the *kcnb2* and *hsp40*, which are upstream and downstream of *TRPA1a*, respectively. The *TRPA1b* gene, however, duplicated multiple times within the Antarctic clade; we found evidence of at least two independent duplications of *TRPA1b* in *H. antarcticus* and *P. georgianus*. We also found a partial duplication of the first half of the *TRPA1b* gene in *C. aceratus*, which clustered independently of the other duplications, and another independent complete duplication of *TRPA1b* in the genome of *D. mawsoni*. All duplicates were annotated *TRPA1b2*. However, the *D. mawsoni TRPA1b2* gene was manually put together from pieces found across six scaffolds (as was the single copy of *TRPA1b* in *N. coriiceps*). This lowered our confidence in the exact number of duplication events. The high variation in copy number and poor assembly quality in this region may be attributable to a high percentage of repetitive sequences in the region around *TRPA1b* in Antarctic notothenioid species. We found that non-Antarctic *C. gobio* and *E. maclovinus* had relatively low repetitive content (21.5% and 13.7%, respectively) in the *TRPA1b* chromosomes or scaffolds. In contrast, the cryonotothenioids had high repetitive content in the regions around *TRPA1b*, particularly in those species that showed evidence of a duplication (*D. mawsoni*: 33.1%; *H. antarcticus*: 54.1%; *C. aceratus*: 72.8%; *P. georgianus*: 64.4%). Cryonotothenioid species with only one copy of *TRPA1b* had variable repetitive content (*N. coriiceps*: 27.9%, *T. bernacchii*: 26.0%, *P. charcoti*: 35.9%, *G. acuticeps*: 64.2%). This increase in repetitive content in cryonotothenioids is due primarily to an increase in DNA transposons, in particular the hobo-Activator transposon (see supplementary table S4, Supplementary Material online).

### Trigeminal Ganglion Transcriptomes

**Fig. 5.**
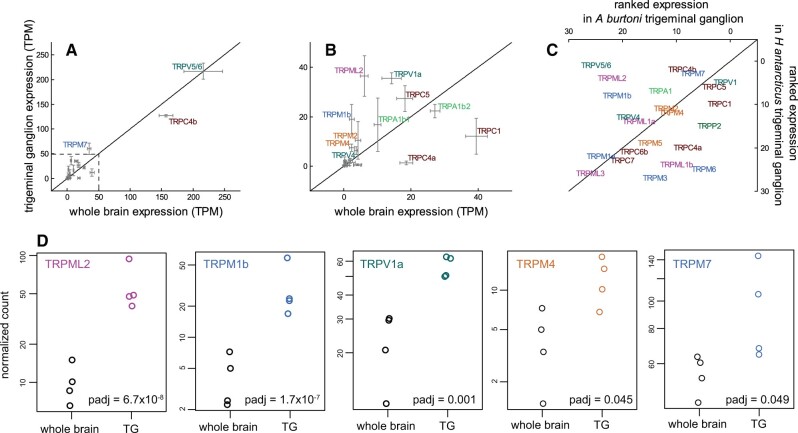
Gene expression data comparing (*a*, and inset expansion *b*) TRP channel expression in the *Harpagifer antarcticus* trigeminal ganglion and whole brain and (*c*) ranked expression levels in *Astatotilapia burtoni* and *H. antarcticus*. Expression levels are in transcripts per million (TPM). Diagonal lines indicate equal expression levels in both comparisons, genes above the line in (*a*) and (*b*) have higher expression in the trigeminal ganglion relative to the whole brain or, as in C, higher ranked expression in *H. antarcticus* compared with *A. burtoni*. (*d*) The five TRP channels with significantly higher expression in the *H. antarcticus* trigeminal ganglion (TG) compared with the whole brain based on counts normalized in DESeq2, adjusted *P* values shown. Colors indicate gene subfamilies, as in figure 2.

We generated mRNA expression data for the trigeminal ganglion and whole brain of *H. antarcticus* (fig. 5). We expected TRP channels involved in thermosensation to have higher expression in the sensory nerves, such as the trigeminal ganglia, relative to the whole brain. We selected trigeminal ganglia as it innervates the face and is relatively large as compared with dorsal root ganglia. As these nerves are closely associated with other cranial ganglia, we confirmed dissection samples contained primarily trigeminal ganglion using correspondence and principal components analyses (supplementary fig. S4, Supplementary Material online).

From highest to lowest expression, the TRP channel genes found in the *H. antarcticus* trigeminal ganglion were *TRPV5/6*, *TRPC4b*, *TRPM7*, *TRPML2*, *TRPV1a*, *TRPC5*, *TRPA1b2*, *TRPM1b*, *TRPA1b1*, *TRPC1*, *TRPM2*, *TRPM4*, and *TRPV4* (fig. 5). Out of 65,459 total transcripts with a nonzero read count, 10.0% (6,576 transcripts) were significantly differentially expressed between the whole brain and the trigeminal ganglia, 26.2% (17,176 transcripts) were not significantly differentially expressed between the two tissues, and 63.3% (41,495 transcripts) were excluded due to low counts. Specifically, 4.5% (2,929 transcripts) had higher expression in the whole brain relative to the trigeminal ganglia, and 5.6% (3,647 transcripts) showed higher expression in the trigeminal ganglia. A total of 9 out of 29 TRP channel genes were significantly differentially expressed between the trigeminal ganglion and the whole brain. *TRPML2* (log_2_ fold change = 2.59; adjusted *P* value = 6.7 × 10^−8^), *TRPM1b* (log_2_ fold change = 3.02; adjusted *P* value = 2.8 × 10^−5^), *TRPV1a* (log_2_ fold change = 1.33; adjusted *P* value = 0.0010), *TRPM4* (log_2_ fold change = 1.67; adjusted *P* value = 0.045), and *TRPM7* (log_2_ fold change = 0.80; adjusted *P* value = 0.049) all had higher expression in the trigeminal ganglion relative to the whole brain. The genes with higher expression in the whole brain compared with the trigeminal ganglion were *TRPC4a* (log_2_ fold change = −3.82; adjusted *P* value = 1.7 × 10^−7^), *TRPC7* (log_2_ fold change = −3.60; adjusted *P* value = 0.017), *TRPC6b* (log_2_ fold change = −2.62; adjusted *P* value = 0.037), and *TRPC1* (log_2_ fold change = −1.69; adjusted *P* value = 0.042; supplementary fig. S5, Supplementary Material online).

We also generated expression data for the trigeminal ganglion of *Astatotilapia burtoni*, a tropical African cichlid (fig. 5 and supplementary fig. S4, Supplementary Material online). In the *A. burtoni* trigeminal ganglion *TRPC1*, *TRPV1a*, and *TRPC5* were the TRP channels with the highest expression (supplementary fig. S6, Supplementary Material online). *TRPV5/6*, *TRPML2*, *TRPM1b*, *TRPC4b*, *TRPM7*, and *TRPV4* all had higher ranked expression in *H. antarcticus* compared with *A. burtoni*.

### Sites under Diversifying Selection

**Fig. 6.**
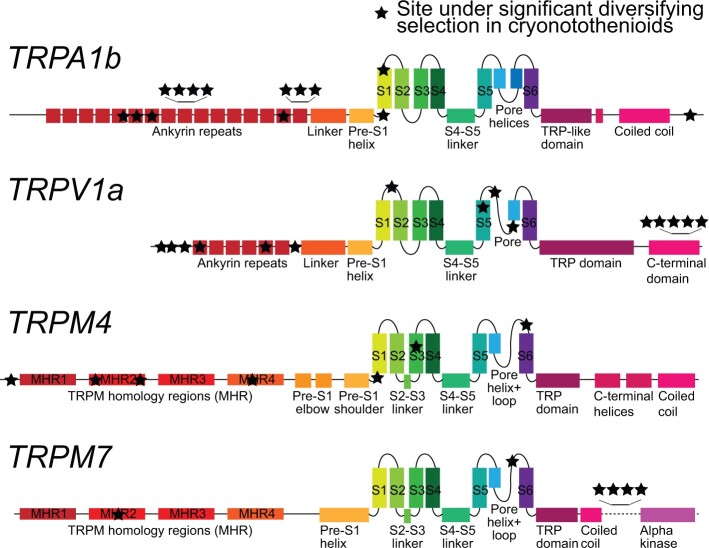
Schematics of selected TRP channels with sites under significant positive selection. Black stars indicate location of sites as determined by the location of the homologous site on channel from a species for which a structure has been determined. TRPA1b site location determined from human TRPA1 (UniProtKB-O75762; schematic adapted from Paulsen et al. 2015). TRPV1a site location determined from human TRPV1 (UniProtKB-Q8NER1; schematic adapted from Liao et al. [2013]). TRPM4 site location determined from human TRPM4 (UniProtKB-Q8TD43; schematic is adapted from Autzen et al. [2018]). TRPM7 site location determined from human TRPM7 (UniProtKB-Q96QT4; schematic is adapted from the mouse channel in Duan et al. [2018]). See supplementary figure S7, Supplementary Material online for the alignments of these sites.

Given the results above, we selected TRPV1a, TRPA1b, TRPM4, and TRPM7 as our top candidates for putative thermosensitive channels in cryonotothenioids and investigated each for site-specific evidence of diversifying selection. At the suggested significance level of *P* < 0.1, we found a total of 74 significant sites in TRPV1a (4.9% of total possible sites in the alignment), 62 sites in TRPA1b (4.9%), 46 in TRPM4 (3.1%), and 124 in TRPM7 (4.8%; supplementary table S5, Supplementary Material online). Each site was inspected to determine which branches of the gene tree were contributing to the signal and only sites where the signal was primarily within the cryonotothenioid clade are shown in the schematic of figure 6 and the alignment in supplementary figure S7, Supplementary Material online. This was a total of 15 sites in TRPV1a, 14 sites in TRPA1b, 7 sites in TRPM4, and 6 sites in TRPM7. According to SIFT, which predicts if substitution have consequences for protein function, all sites except the most C-terminal site in TRPV1a were predicted to be “tolerated,” or nondeleterious, with a SIFT score >0.05 (Sim et al. 2012; supplementary table S5, Supplementary Material online).

## Discussion

We found notothenioid fish genomes have between 23 and 29 TRP channel genes, similar to other teleosts. Within the Antarctic clade—the cryonotothenioids—we found multiple, independent duplications of *TRPA1b*. We hypothesized that TRP channels functioning as temperature sensors in cryonotothenioid fishes would demonstrate enrichment in the sensory nerves and evidence of changes in selective pressure coinciding with adaptation to the frigid Southern Ocean. Based on this, we identified multiple candidate channels: *TRPA1b*, *TRPV1a*, *TRPM4*, and *TRPM7*, and explored these channels further with a site-specific analysis.

The neural encoding of environmental temperature in organisms is redundant: temperature-gated channels overlap in activity range, and single neurons express multiple channels (Gallio et al. 2011; Vandewauw et al. 2018). TRP channels also form heteromers with distinct characteristics within and across subfamilies (Cheng et al. 2012; Gees et al. 2012). Further, the activity of both cold-activated and warm-activated channels can contribute to neural signals at the organismal level (Paricio-Montesinos et al. 2020). We conducted a comprehensive and unbiased exploration of all TRP channel genes in Antarctic notothenioid fish. Other channels, however, may contribute to vertebrate sensation of peripheral temperature, and those were not explored here (Hoeffstaetter et al. 2018; Buijs and McNaughton 2020; MacDonald et al. 2020).

### TRP Channel Evolution

The complement of TRP channel genes found in notothenioids was similar to other teleosts and supported recent analyses of TRP evolutionary history. Notothenioids and other teleosts have a single *TRPV5/6* ortholog, which appears to be the ancestral condition (Flores-Aldama et al. 2020). All notothenioid species studied had two *TRPV1* paralogs, due to the teleost ancestral genome duplication (supplementary fig. S3, Supplementary Material online; Saito et al. 2011). All teleosts lack *TRPV2*, *TRPV3*, and *TRPM8* (Saito and Tominaga 2015); we also found no evidence of *TRPC3* in notothenioids, however, this gene is present in at least some other teleosts. In the protein tree, TRPM2, TRPM4, and TRPM5 clustered separately from TRPM1a, TRPM1b, TRPM3, TRPM6, and TRPM7 in support of the hypothesis that there were two ancestral TRPM clades in metazoans: αTRPM and βTRPM (Himmel et al. 2020). We found no evidence of *TRPN* in notothenioids and only a single *TRPP* gene (*TRPP2*).

### TRPM1b and TRPML2

TRPM1b and TRPML2 had the highest expression in the sensory nerves relative to the whole brain, but in different parsings of the data, there was not a consistent signal of intensified selection in cryonotothenioids. TRPML2 and all TRP mucolipins are generally intracellular (Gees et al. 2012). In zebrafish, TRPM1b is expressed in the retina and functions in bipolar cells in human light perception (Kastenhuber et al. 2013).

### Investigating Residues under Diversifying Selection

Physiological processes, such as sensation, are expected to evolve via structural rather than regulatory evolution (Rosati and McKinnon 2009). Adapting to cold environments seems to require mutations to the primary amino acid sequence that result in changes to desensitization, activity, kinetics, and/or shifted dynamic range (Lynch et al. 2015; Laursen et al. 2016; Saito et al. 2016; Matos-Cruz et al. 2017; Saito and Tominaga 2017; Luo et al. 2019; Saito et al. 2019; Hori and Saitoh 2020; Yang et al. 2020). Our comparison of ranked TRP expression between Antarctic *H. antarcticus* and tropical *A. burtoni* demonstrates that our top candidates for thermosensation have similar ranked expression between the two species in support of the hypothesis that it is primarily structural evolution of existing thermosensors that underlies adaptation to new thermal environments. However, existing sequencing data are currently insufficient to investigate regulatory changes in thermosensation within notothenioids, both across species and ontogeny. Further, in this study, we investigated changes in selective pressure only on the protein coding sequence. It has been proposed that 3′ UTRs in particular may be a target of thermal adaptation (Somero 2020).

In assessing the significance of specific substitutions, we relied on sequence homology primarily with humans and rodents. We specifically discuss sites that showed evidence of significant diversifying selection in the ancestral cryonotothenioid branch, that is, those sites that arose in the same evolutionary period as antifreeze proteins. We otherwise did not restrict our analysis as sites across TRP channel proteins are known to modulate temperature gating (Voets 2012).

### TRPM4: A Promising Candidate with Limited Data

TRPM4 is high on the list of candidate channels, not only because it was found to be under significantly intensified selection and also enriched in the trigeminal ganglia, but also because it is related to TRPM8—a channel which, in mammals, is activated by relatively low temperatures (<26 °C; Peier et al. 2002; Bautista et al. 2007). Of course, such “cool” temperatures are lethally hot for cryonotothenioids and, further, *TRPM8* has been lost in teleost fish (Saito and Shingai 2006; Himmel et al. 2020). *TRPM4* has been elaborated in teleosts, and some species have up to four paralogs; however, we found only one in notothenioids (Kastenhuber et al. 2013). In mammals, TRPM4 gating appears to be modulated by heat, but data are limited to a single study (Talavera et al. 2005). In teleosts, TRPM4 is associated with learning in response to light stimuli and inflammation, but is otherwise uncharacterized (You et al. 2020; Li et al. 2021).

Most of the sites under significant diversifying selection in cryonotothenioid TRPM4 are found in the N-terminal TRPM homology regions (MHRs), which serve several purposes including subunit interaction, ligand binding, and signal transduction to the pore (Huang et al. 2020). In human TRPM4, previous work has shown the G325A mutation, but not neighboring mutations, eliminates ATP modulation of calcium-induced desensitization (Nilius et al. 2005). Cryonotothenioids specifically have an S at this residue, which may affect ATP binding. Another cryonotothenioid-specific serine substitution (ancestral appears to be valine) is of note for its location in the extracellular pore loop, one of the last residues before the S6 helix. In TRPM8, reducing the hydrophobicity of sites in the pore region that shift in exposure to water upon gating diminishes channel thermosensitivity and has been proposed as a mechanism of dampening cold pain for organisms adapted to cold environments (Yang et al. 2020).

Although mammalian TRPM4 is not calcium permeable, it is directly activated by intracellular calcium (Vangeel and Voets 2019). One significant site in the middle of the S3 is just to the extracellular side of the calcium-binding site and is substituted to a valine in cryonotothenioids (Autzen et al. 2018). A further site is found just before the S1, a region which is intrinsically disordered in human TRPM4. No sites were found under significant diversifying selection in the C-terminal domain, which is relevant for the hypothesis suggesting folding and unfolding of the C-terminal underlies temperature-dependent gating in TRPM8 (Díaz-Franulic et al. 2020).

### TRPM7: A Potential Molecular Basis for Unusual Magnesium Homeostasis in Notothenioids

TRPM7 was found to be significantly upregulated in the sensory ganglia relative to the whole brain and showed evidence of significantly intensified selection in cryonotothenioids. It also was our only candidate gene that had higher ranked expression in the Antarctic fish as compared with the tropical fish. Although several members of the TRPM subfamily are thermosensitive in other species, those that are thermosensitive are descended from the ancestral βTRPM gene while TRPM7 is descended from the αTRPM (Himmel et al. 2020). However, given the evidence, we determined a site-specific analysis would be worthwhile.

Six residues were found to be under significant diversifying selection in cryonotothenioid TRPM7. Four of these are found in a region between the C-terminal coiled-coil domain and the alpha kinase which has not been resolved in any structure (Duan et al. 2018; Huang et al. 2020). These sites are within a serine/threonine-rich region identified in mice as containing many phosphorylation sites that control the activity of the alpha kinase (Clark et al. 2008). Two of the cryonotothenioid specific-substitutions are serines, suggesting that these residues might be phosphorylation sites (the others are a proline/arginine and a cysteine). In mice, the kinase domain can be cleaved from the channel, and, whereas not required for channel function, it modulates channel magnesium sensitivity (Matsushita et al. 2005; Krapivinsky et al. 2014). In addition, recent work shows magnesium-sensitivity of human TRPM7 is modulated by oxidative stress via certain cysteine residues (Inoue et al. 2021). Notothenioids are noted as having higher urinary magnesium concentrations than any other teleost, as high as 417 millimoles per liter in *Trematomus bernacchii* but more typically closer to 200 millimoles per liter (Dobbs and DeVries 1975). Notothenioids have aglomerular kidneys to retain their antifreeze proteins and thus rely on active ion secretion (DeVries 1988). They maintain serum magnesium at levels similar to other teleosts—against a 100-fold concentration gradient. Dobbs and DeVries (1975) speculate that this increased magnesium load could either be due to higher rates of drinking seawater and/or increased magnesium permeability at the gut. Petzel (2005) found notothenioids actually have low drinking rates compared with other teleosts. O’Grady et al. (1982) measured lower concentrations of magnesium in the intestinal fluid of notothenioids relative to temperate teleosts. As TRPM7 is an important regulator of magnesium homeostasis in mammals (Schmitz et al. 2003), these residue substitutions might be a key molecular mechanism involved in either magnesium permeability at the gut or maintaining a steep gradient at the kidney.

The two other cryonotothenioid-specific significant substitutions in TRPM7 are in the middle of the second TRPM homology region (MHR) and in the extracellular region just after the pore loop before the S6. The site in the pore (S1060 in humans and mice and a valine in most cryonotothenioids) is found where the radius of the pore is restricted, so it might be involved in ion selectivity or binding (Huffer et al. 2020).

We conclude TRPM7 is likely not involved in thermosensation, but hypothesize that it may underlie the unusual magnesium levels maintained in notothenioid urine. Recently, TRPM7 has been shown to sense fluid shear stress, which may explain its high expression in the sensory nerves (Yankaskas et al. 2021).

### TRPV1a: A Likely Thermosensor Candidate

*TRPV1a* had significantly higher expression in the trigeminal ganglion relative to the whole brain and some evidence of intensified selection within the cryonotothenioid clade. TRPV1 has been shown to mediate behavioral responses to heat in the range of 28–35 °C in zebrafish embryos and is activated by heat in every vertebrate characterized so far (Gau et al. 2013; Saito and Tominaga 2017).

In the site-specific analysis, residues across the protein showed evidence of significant diversifying selection in the common ancestor of cryonotothenioids. Six N-terminal sites were significant, including two sites in the ankyrin repeats. One (D114 in humans, glycine in cryonotothenioids, threonine in other teleosts) is just upstream of a site found to reduce thermosensitive gating in mammals adapted to extreme thermal environments (Laursen et al. 2016). Five sites within a ∼20-residue segment of the C-terminal domain of TRPV1a were identified as being under significant diversifying selection within cryonotothenioids. Deletion of a larger region including this segment lowers the thermal activation threshold in TRPV1 from rat (*Rattus norvegicus*; Prescott and Julius 2003). In vampire bats (*Desmodus rotundus*), a TRPV1 isoform specifically expressed in infrared-detecting sensory neurons is spliced to exclude this region and has a thermal activation threshold 10 °C lower than the longer isoform found in other sensory neurons (Gracheva et al. 2011).

These C-terminal residues as well as three of the N-terminal cryonotothenioid substitutions are found in regions of the TRPV1a protein that are thought to be intrinsically disordered. Disordered regions do not resolve in structures, but are likely key modulators of channel function and are posited to be involved with shifting thermal activation threshold to physiologically relevant temperatures in TRPV thermoTRPs (Goretzki et al. 2021).

The pore region of TRP channels has been proposed to be intrinsically heat sensitive and important for stabilizing heat-induced opening (Grandl et al. 2010; Zhang et al. 2017). Two sites in the pore region of the TRPV1a channel showed evidence of significant diversifying selection. The first (residue C621 in human TRPV1, V591 in *E. maclovinus*, substituted to an isoleucine in all cryonotothenioids) is within the pore turret (Yang et al. 2010). The pore turret is a particularly mobile region on the extracellular side of the protein and the turrets of each subunit move closer to each other upon temperature activation but not ligand-mediated activation (Yang et al. 2010; Cui et al. 2012). Mutations in the turret have been tied to shifting thermal thresholds in mammalian TRPV1 (Du et al. 2020). The second residue (N629 in humans, S604 in *E. maclovinus*, substituted to either K or T in several cryonotothenioids) is just before the pore helix within the pore forming domain. Mutating this residue to K in rats has been shown to blunt temperature, but not ligand, responses by decreasing the probability of long open times (Grandl et al. 2010).

### TRPA1b: Multiple Cryonotothenioid Duplications and Significant Residues in the Ankyrin Repeat Domains

We found evidence of up to four independent duplications of *TRPA1b* within cryonotothenioids; each duplication appeared to be species specific. We named these paralogs *TRPA1b1* and *TRPA1b2*. We posit that multiple duplications could be due to high repetitive content in the region around this gene. Across the genome, there is evidence of a burst of transposable elements in the DNA of cryonotothenioids but not basal temperate notothenioids (Auvinet et al. 2018; Chen et al. 2019; Kim et al. 2019; Bista et al. 2020). We found enrichment of DNA transposons in the scaffolds associated with *TRPA1b* duplicates compared with species with no duplication. Our certainty of the exact number of duplications is limited because some copies were not found continuously on the same scaffold. To clarify this, long read sequencing is needed for repetitive regions; currently standard NextGen methods likely underestimate repetitive content (Baalsrud et al. 2018; Bargelloni et al. 2019). Tandem repeats and transposable elements are particularly susceptible to sequencing errors, a common issue in teleosts (Tørresen et al. 2019).

Although both *TRPA1b* paralogs were expressed in the trigeminal ganglion of *H. antarcticus*, neither gene showed significant enrichment of expression relative to the brain. However, because of the duplications and evidence of significantly intensified selection, we investigated this gene for sites under diversifying selection. TRPA1 is a temperature sensor in both endotherms and ectotherms, but whether it is a cold or a heat sensor varies depending on species (Laursen et al. 2015). There is evidence that TRPA1 is gated by heat in medaka, and both heat and cold in zebrafish and *Takifugu* (Oda et al. 2016, 2017, 2018). *TRPA1* knockout mice have significantly reduced cold nociception (Karashima et al. 2009). In snakes, *TRPV1* is under consistent purifying selection, whereas *TRPA1* shows evidence of positive selection and varies in thermal threshold to enable hunting by sensing infrared radiation (Gracheva et al. 2010; Geng et al. 2011).

Generally, *TRPA1* has higher sequence divergence across vertebrates compared with other TRP genes, particularly in the extracellular loops (Saha et al. 2019). It has been hypothesized that these extracellular regions have diverged to adapt to each specific environment (Saha et al. 2019). However, we found only one extracellular site under significant diversifying selection in cryonotothenioids. This site is located in a particularly divergent extracellular loop between the first and second transmembrane domains (N739 in humans, methionine in notothenioids).

Most of the significant TRPA1 sites are in the ankyrin repeat domains, which have been specifically tied to shifting thermal threshold via positive selection in infrared detecting snakes, although none of the specific sites were shared between these analyses (Cordero-Morales et al. 2011; Geng et al. 2011). One site tied to high thermal sensitivity in snakes (S428 in humans and T434 in rattlesnake) showed a notothenioid-specific cysteine substitution but was not found to be significant in our analysis (Yokoyama et al. 2011). Another site in ankyrin repeat 6 (R or K in other vertebrates, K256 in humans, T in notothenioids) is right next to a site that has been proposed to switch mouse TRPA1 from being cold activated to being heat activated (Jabba et al. 2014).

Of the remaining two significant sites in TRPA1, one was found in the pre-S1 helix, which is thought to be important for electrophile sensing (Paulsen et al. 2015). Notothenioids retain two out of the three cysteines important for allyl isothiocyanate sensitivity, similar to other teleosts (Hinman et al. 2006).

### TRP Channel Gating and Cold-Adapted Proteins: Why This Study Matters for Biophysics

By identifying a channel that might gate closer to 0 °C than any channel characterized to date, we hope to provide insight into thermosensitive gating mechanisms of TRP channels. The mechanism of temperature-dependent gating remains under debate. Hypotheses include 1) a large difference in activation energy between channel opening and closing (Voets et al. 2004), 2) a large difference in heat capacity between the open and closed channels (Clapham and Miller 2011), and 3) part of the protein acts as a temperature sensor and is allosterically coupled to channel gating (Jara-Oseguera and Islas 2013). These hypotheses are not mutually exclusive and may be occurring to various extents depending on the particular channel. For example, recent work has indicated that folding and unfolding of certain areas of the channel with gating is associated with thermosensitivity, in support of both hypotheses (2) and (3) (Sánchez-Moreno et al. 2018; Díaz-Franulic et al. 2020). Hypothesis (2) predicts that channels should be open at both sufficiently high and low temperatures, as long as the change in heat capacity itself does not depend on temperature (Yeh F, personal communication), yet this phenomenon has not been measurable in most channels as the low temperature gating threshold is predicted to occur far below the freezing point of water.

A cold-sensitive TRP channel must not only gate in response to lower temperatures but also have a robust dynamic range of activity within the range of ecologically relevant temperatures; it is unclear how this might occur. Studies on protein adaptations from metazoans living below 0 °C are limited, and further, the majority of the work examining how proteins adapt to cold environments has focused on globular enzymes (Gulevsky and Relina 2013; Berthelot et al. 2019). These studies consistently find cold-adapted proteins have decreased hydrophobicity, lowered activation energy, and increased flexibility (Marshall 1997; Hochachka and Somero 2002; Roulling et al. 2011). This makes identifying a TRP channel that gates at temperatures near freezing compelling for three thermodynamic reasons: 1) a large change in enthalpy during gating is required to make gating thermosensitive, but cold-adapted proteins have low enthalpy to minimize activation energy (Feller and Gerday 2003; Roulling et al. 2011; Ito et al. 2015); 2) there is a potential conflict in selective pressures, given that hydrophobic interactions are reduced at low temperatures due to diminished entropy, but thermosensitive gating of TRP channels is particularly sensitive to reductions in residue hydrophobicity (Privalov 1990; Sosa-Pagán et al. 2017); 3) molecular motion in cold environments is due to changes in hydration of residues rather than conformational entropy, and changes in residue hydration have been posited as the mechanism of temperature gating in TRP channels (Feller 2010; Clapham and Miller 2011). Therefore, the channels identified in this study provide a powerful comparison for investigating biophysical explanations for thermosensitive gating and cold adaptation.

The trade-off for increased molecular flexibility is reduced stability at all temperatures (Feller 2010; Temussi 2011; Santiago et al. 2016). For channels, the primary challenge with retaining function at low temperatures is likely to be maintaining stability of quarternary structure (Bock and Frieden 1978; Privalov 1990; Marshall 1997). High latitude notothenioids also have high concentrations of solutes, which are destabilizing to proteins (Dobbs and DeVries 1975; Yancey et al. 1982). To counteract this, they have constitutively high expression of molecular chaperones and high trimethylamine N-oxide (Somero 2020; Raymond and DeVries 1998). Membrane proteins, such as TRP channels, also have lipid–protein interactions and it has been suggested that these interactions may determine thermal limits of activity (Haschemeyer 1980; Marshall 1997; Weinstein and Somero 1998; Galarza-Muñoz et al. 2011). Together, this makes TRP channels from cryonotothenioids a fascinating case, and this study lays the groundwork for answering long-standing questions about temperature gating and cold function in biophysics, thermodynamics, and evolution in the cold. In addition, cold-adapted proteins have extensive uses in industrial applications (Santiago et al. 2016).

### Cold Is Not the Only Selective Pressure

Given the polymodal nature of TRP channels, they often act as physiological coincidence detectors for multiple stimuli (Zheng 2013). Although low temperature may be a strong selective pressure for organisms in the Southern Ocean, this habitat has many other unique biotic and abiotic factors that drive the evolutionary strategy of cryonotothenioids (Eastman 2000). This strategy involves a cascade of behavioral, physiological, molecular, and genetic adaptations and has enabled them to diversify so successfully. TRP channels are likely to be components of several of these adaptations.

Reactive oxygen species (ROS), for example, are considered to be major stressors for organisms in the highly oxygenated Southern Ocean waters. Evidence suggests the primary selective pressure for Antarctic bacteria is oxidative stress (Russo et al. 2010). Cryonotothenioids express high levels of antioxidants but still show evidence of high oxidation stress (Todgham et al. 2007; Chen et al. 2008; Peck 2016, O’Brien et al. 2018). Several TRP channels are gated or modulated by ROS, including mammalian TRPV1, TRPA1, and TRPM7 (Takahashi et al. 2011; Inoue et al. 2021; Pantke et al. 2021). It has been hypothesized that aversive behavioral responses to heat are actually mediated by ROS gating of TRP channels (Arenas et al. 2017). Typically, redox sensitivity is modulated by methionine or cysteine residues, and methionines are overrepresented in the proteins of notothenioids (Berthelot et al. 2019). In TRPV1, cryonotothenioids and other teleosts lack two of the N-terminal cysteine residues important for oxidant-induced gating in mammalian channels, but do maintain one C-terminal cysteine shown to be important in redox sensitivity (Chuang and Lin 2009; Pantke et al. 2021). Notothenioid TRPA1b retains five out of ten cysteines that affect redox gating in mammalian channels, similar to other teleosts (Takahashi et al. 2011). In TRPM7, both cysteines important for redox sensitivity in the human channel are conserved in cryonotothenioids, and we found significant diversifying selection at an additional cysteine substitution specific to cryonotothenioids (Inoue et al. 2021). As oxidants can potentiate heat-evoked responses via these residues, the loss or gain of cysteines may contribute to changing temperature gating in the presence of oxidants (Pantke et al. 2021).

As another example, notothenioids are descended from a benthic common ancestor and do not have a swim bladder, yet the clade has undergone “depth-related diversification” meaning several species secondarily occupy the water column (Eastman 2005). To increase buoyancy without a swim bladder, these species have reduced skeletal ossification and increased lipid deposition (Matschiner et al. 2015; Daane et al. 2019). TRPV4 is hypothesized to be involved in mediating calcium deposition in bones in response to mechanical stress (Nilius and Owsianik 2010). TRPV4 also mediates differentiation of adipocytes, chondrocytes, and osteoblasts/osteoclasts in humans, and *TRPV4* inactivation mutations reduce calcium uptake (Kang et al. 2012). In this study, we found that selection was significantly relaxed on cryonotothenioid *TRPV4* relative to other teleosts, suggesting a potential molecular mechanism in which reduced *TRPV4* expression and functionality allowed for the accumulation of nonsynonymous mutations, or vice versa, as has been shown in other genes related to bone density in notothenioids (Daane et al. 2019).

### Conclusion and Consequences

We conclude that TRPV1a, TRPA1b, and TRPM4 are the best candidate proteins for thermosensors in cryonotothenioids and provide the groundwork for further investigation including functional characterization. Cryonotothenioids are extreme thermal specialists; unfortunately, climate warming is expected to result in the decline of specialist species at the cost of ecosystem services and diversity of biological function (Clavel et al. 2011). The frequency and intensity of marine heatwaves is increasing (Frölicher and Laufkötter 2018). Given that marine species fill their fundamental thermal niche more completely, shift distribution more rapidly, and are more vulnerable to warming than terrestrial species, studies on how notothenioids cope with changes in their thermal environment over the next few decades will be informative for this new era of climate adaptation (Donelson et al. 2019; Pinsky et al. 2019). Evidence indicates there were reduced population sizes of notothenioids during eras of high thermal variability (Kim et al. 2019). Given that notothenioids are key to the Southern Ocean food web (McMullin et al. 2017), the future survival and distribution of these fish may be a determining factor for the future structure of the Antarctic biome.

## Materials and Methods

### Transcriptome Mining

Transcriptomic data for several notothenioids were collected (supplementary table S1, Supplementary Material online). Transcriptome databases were generated for each species and then queried using command line BLAST (TBLASTN) on the Texas Advanced Computing Center (TACC) terminal (Altschul et al. 1997; Camacho et al. 2009). We found TBLASTN was sufficiently sensitive (yielding 10–150 hits per gene) without being computationally unwieldy downstream (e.g., TBLASTX yields 200–1000+ hits per gene). Query files included protein reference sequences from *Homo sapiens*, *Mus musculus*, *Xenopus tropicalis*, *Gallus gallus*, *Oreochromis niloticus*, *Oryzias latipes*, *Danio rerio*, and *Gasterosteus aculeatus*, or the available subset of those species (see supplementary tables S1 and S2, Supplementary Material online). The ten mRNA sequences with the lowest *e*-value were returned for each alignment, and if necessary, translated using TransDecoder (v 5.5.0, retrieved from github.com/TransDecoder/TransDecoder/wiki, last accessed 1/26/22). These sequences were then combined with all the reference protein sequences and aligned using MAFFT with the fast and progressive method (v 7.490; Katoh and Standley 2013). MAFFT was chosen after comparing trees built from alignments with MAFFT, Clustal Omega (Sievers and Higgins 2018), and MUSCLE (Edgar 2004). Trees had a different topology depending on the alignment used, but those built using MAFFT alignments clustered the proteins most accurately with a minimal number of very short sequences. Initial trees were built in this way for all hits from each notothenioid transcriptome using RAxML-ng (v 0.9.0; Kozlov et al. 2019), using the LG substitution model and the –search1 option, which returns a maximum likelihood tree from a single random starting tree (Le and Gascuel 2008). In testing, trees built using this option had identical topology to the best scoring topology generated from the default search starting with ten random and ten parsimonious trees. BLAST hits that did not cluster with reference TRP channels were excluded and those that did cluster were given preliminary annotations. All sequences with these preliminary annotations from all notothenioid databases were then combined with reference protein sequences and realigned using MAFFT. ModelTest-ng (v0.2.0; Flouri et al. 2015; Darriba et al. 2020) determined the substitution model with the highest likelihood was JTT+G4+F (log likelihood -293469). A final tree was built using RAxML-ng with default parameters and a JTT+G4+F substitution model. As a support metric, 200 bootstrap replicates were conducted using the –bootstrap option in RAxML-ng. Predicted protein sequences were never trimmed during this analysis, but if fragments clearly appeared in alignments to be sequential and nonoverlapping, they were collated (indicated by “+” in accession files; supplementary tables S1 and S2, Supplementary Material online).

### Tests for Intensified or Relaxed Selection

Transcriptomic sequences corresponding to sequences verified in the protein tree were uploaded to Translator-X (accessed from translatorx.co.uk, last accessed January 26, 2022; Abascal et al. 2010), translated, and protein sequences were aligned using MAFFT (Katoh et al. 2005). The protein alignments were then trimmed to remove poorly aligned sites using GBlocks with either all options for a “less stringent” selection selected or none of these options (“more stringent”; Castresana 2000). The trimmed protein alignments were then reverse translated back to mRNA. Maximum likelihood trees were inferred among these trimmed transcript alignments using RAxML-ng with a GTR+FO evolutionary substitution model and nonteleost reference sequences as the outgroup. The tree with the highest likelihood was selected among maximum likelihood trees generated from ten parsimonious and ten random starting trees using RAxML-ng. The trees were combined with the trimmed and aligned mRNA sequences and tested for evidence of selection using HyPhy (v 2.5; Kosakovsky Pond et al. 2020).

Specifically, each gene was tested for selection intensification or relaxation using RELAX; this program compares nonsynonymous/synonymous substitution ratio (d*N*/d*S* or *ω*) distributions in reference branches with the d*N*/d*S* distributions sites in the test branches by classifying sites into three bins based on d*N*/d*S* (*ω*_1_, *ω*_2_, and *ω*_3_) and comparing the shift in those *ω* classes in the test branches relative to the reference branches (Wertheim et al. 2015). Depending on the direction of that shift, RELAX infers selection relaxation, intensification, or neither (no change).

All cryonotothenioid branches and associated nodes were selected as the test branches, and all other branches and nodes (including reference species and non-Antarctic notothenioids) were used as the reference set. For each gene the RELAX analysis was run thrice with 1) all available data and less stringent alignment trimming, 2) selected data (most complete transcripts) and less stringent trimming, and 3) selected data with more stringent alignment trimming. Substitution ratio metrics were verified using the PAML package with default options and an initial omega value of 1 (v 4.9j; Yang 2007).

### Genome Mining

To determine gene gain or loss, we had access to seven notothenioid genomes representing six families (see supplementary table S1, Supplementary Material online for species and sources). The corresponding transcriptomes for six of these genomes were included in the transcriptome analysis. To investigate whether notothenioids have lost or gained TRP channel genes, we queried each genome with the TRP mRNA sequences identified in the transcriptome analysis using command line BLAST (BLASTN). If genes were not represented in the transcriptome for that particular species, mRNA sequences from a different species were added to the query (e.g., all the *TRPP* subfamily, *TRPC3*, and *TRPN1*). For *Cottoperca gobio*, the only genome for which no transcriptome was available, a query file of available complete transcripts from other species was used (primarily *Gasterosteus aculeatus*, *Mus musculus*, and *N.coriiceps*). All hits were returned, and inspected for chromosomal or scaffold location and completeness. If one mRNA sequence returned multiple complete sequence alignments from different locations in the genome, that gene was considered to have multiple copies. Sequences were aligned using MAFFT, and maximum likelihood gene trees were built using RAxML-ng, as described, to determine if duplications arose independently. For the *TRPA1* gene tree 500 bootstrap trees were inferred for support. If no hits were returned for a sequence, the deletion was investigated and confirmed by searching for genes found in synteny in related species.

We used RepeatMasker to inspect the regions around *TRPA1b* for repetitive content (4.1.1; Smit et al. 2015). We input all scaffolds or chromosomes from our genomic comparison species that contained pieces of *TRPA1b* and identified the sequences as “notothenioidei” within the Dfam database (release 3.3; Storer et al. 2021).

### Trigeminal Ganglion Transcriptomes

To identify the genes expressed in the sensory ganglia, five *Harpagifer antarcticus* (three male, two female) were obtained from the aquarium at the British Antarctic Survey in Cambridge, England. The fish had originally been captured at 2–3 m depth off the coast of Rothera Research Station, Antarctica, in 2002. The fish were held in the aquarium in England at 0 °C until euthanized for this study in June 2018. Individual fish were placed in a solution of MS-222 (approximately 270 mg/ml) until opercular stop (252 ± 14 s) and then for another 10 min, after which the fish were removed from the MS-222 solution and the spinal cord severed. Trigeminal ganglion, pancreas, liver, whole brain, skin, gonads, muscle, intestine, and otoliths were collected and stored in RNAlater at 0 °C for 24 h and subsequently at −80 °C. Trigeminal ganglion was taken as the largest cranial nerve extending rostrally from underneath the hindbrain. Pancreatic tissue was taken as the fatty tissue that connects the triangular shape made by the liver, stomach, and spleen as described in Luchini et al. (2015). Animals were captured, held, and euthanized under permit (33/2017) from the Foreign and Colonial Office of the United Kingdom under the Antarctic Act 1994/Antarctic Act 2013, Antarctic Regulations 1995/490 (as amended).

Four *Astatotilapia burtoni* (all male) were obtained from stocks held at the University of Texas at Austin. Fish were euthanized by severing the spinal cord at the base of the skull, and trigeminal ganglion and pancreas were collected and stored in RNAlater at 4 °C for 24 h and subsequently at −80 °C. Fish were held and euthanized in accordance with the Institutional Animal Care and Use Committee at the University of Texas at Austin (IACUC protocol #AUP-2018-00236).

RNA was extracted from the trigeminal ganglion, pancreas, whole brain, and liver samples of four of the *H. antarcticus* and the trigeminal ganglion and pancreas of all four *A. burtoni* specimens using the RNeasy Mini Kit adding the RNase-Free DNase step (Qiagen, Hilden, Germany). RNA concentrations were estimated using a Nanodrop 2000 spectrophotometer (24 ± 1.7 ng/µl, min = 4.3 ng/µl, max = 39.3 ng/µl). The mRNA was then sequenced by the Genomic Sequencing and Analysis Facility at the University of Texas at Austin. The sequencing was done on an Illumina HiSeq 2500 system using a TagSeq generated library, which targets the 3′ end (Meyer et al. 2011; Lohman et al. 2016). Single-end reads of 100 bp (SR 100) were compiled into FASTQ files (accessible on NCBI Bioproject ID PRJNA758918).

Reads with the same degenerate header and first 20 bp were removed from FASTQ files using TagSeq-specific scripts (tagseq_clipper.pl, last accessed 1/26/22 from https://github.com/z0on/tag-based_RNAseq.git). Then reads were trimmed and quality filtered with cutadapt (-a AAAAAAAA -a AGATCGG -q 15 -m 25; Martin 2011). Reads were then mapped using Bowtie 2 (Langmead and Salzburg 2012). Initially, we mapped the *H. antarcticus* reads to various reference species: *D. mawsoni* (17.8% alignment rate), *N. coriiceps* (55.7% alignment rate), and *C. aceratus* (35.1% alignment rate). These reference transcriptomes were selected because they included the most complete set of TRP channel transcripts. The *H. antarcticus* reference transcriptome (generated from six tissues: brain, white muscle, liver, kidney, skin and heart; Berthelot et al. 2019) contained only 19 of 29 TRP channel genes, and several were incomplete. To remedy this, the missing or incomplete TRP channel transcripts were predicted from *H. antarcticus* genomic data and added to the *H. antarcticus* reference transcriptome prior to remapping. Additionally, because TagSeq is a 3′-targeted sequencing method, the 500 bp immediately following the coding sequence were included as a best guess of the 3′ UTR. The length of 3′ UTRs varies in teleosts, but the median length for nine species in one study was 723 bp (Xiong et al. 2018). The mean length of the 3′ UTRs for the *H. antarcticus* TRP channels that were present in the reference transcriptome was 546 ± 91 bp (max 1324, min 128). Further, expression levels of TRP channels in this study did not depend on the length of the 3′ UTR (data not shown). Each TRP channel sequence in the *H. antarcticus* reference transcriptome was manually checked for completeness, the 3′ UTR, and the presence of other genes in the same transcript sequence. The overall alignment rate of reads to this manually edited *H. antarcticus* transcriptome was 84.6%. *A. burtoni* reads were mapped to *O. niloticus* (76.1% alignment rate; NCBI assembly GCF_001858045.2) and *A. burtoni* (77.8% alignment rate; NCBI assembly GCF_000239415.1). The *A. burtoni* counts were used. TagSeq-specific scripts were used to compile count data. Differential gene expression analysis was done in RStudio (R version 4.0.2) using DESeq2 via Bioconductor (Love et al. 2014; release 3.11). Genes were considered differentially expressed if their *P* value was below 0.05, after adjusting for multiple hypothesis testing using a false discovery rate.

### Dissection Verification of the Trigeminal Ganglion

As *H. antarcticus* is a relatively small species (mean length of sampled individuals 11.9 ± 0.3 cm), we wanted to verify that the dissected and sequenced cranial nerve was, in fact, the trigeminal ganglion. According to Eastman and Lannoo (2004), notothenioid trigeminal ganglia are closely associated with the anterior lateral line nerves as well as cranial nerve VIII. To determine if any or all of these nerves were present in the dissected samples, we compiled lists of genes expressed in each nerve in zebrafish (*Danio rerio*) according to expression data available on the Zebrafish Information Network (ZFIN; Ruzicka et al. 2019). According to these lists, 76 genes are specifically expressed in the zebrafish trigeminal ganglion, 14 in cranial nerve VIII, 8 in the anterior lateral line, and 30 genes had shared expression in at least two of these nerves. We used NCBI-based BLAST to find all of the orthologs for these genes in the genome of *N. coriiceps*. Then we looked for expression of those genes in each dissection sample (using counts mapped to the same *N. coriiceps* reference assembly GCF_000735185.1). We found expression of some tissue-specific genes from all three cranial nerves in our dissection samples. We then performed a correspondence analysis (similar to a principal components analysis but for presence/absence data) to examine which tissue the dissection samples most closely resembled (supplementary fig. S4, Supplementary Material online). The dissection samples clustered together, and as a group they clustered most closely to the trigeminal ganglion of the zebrafish. We therefore conclude that the dissection samples likely contain all three cranial nerves, but include primarily trigeminal ganglion tissue.

### Evidence of Site-Specific Diversifying Selection

TRPA1b, TRPV1a, TRPM4, and TRPM7 were chosen for further analysis on sites in the protein that show evidence of selection. Complete, untrimmed alignments of transcriptomic sequences previously generated with MAFFT, including all reference sequences, were combined with maximum likelihood trees generated with RAxML-ng, as described previously. These files were analysed by command-line MEME (Mixed Effects Model of Evolution, part of the Hyphy package; version 2.1.2; Murrell et al. 2012). MEME looks for signatures of selection on codons and allows nonsynonymous/synonymous substitution rates to vary over branches in the tree. A list of sites with evidence of significant diversifying selection was generated at the suggested significance level of *P* < 0.1. Each site was inspected manually to determine if the branches contributing to the signal were within the cryonotothenioid clade. If so, these sites were mapped onto topological maps of the human or mouse channel as sites of interest. We ran the alignments and significant substitutions through SIFT as a means of predicting if they have deleterious functional consequences (Sim et al. 2012).

### Data Plotting and Statistics

Data were plotted using Origin 2018 (OriginLab, Northampton, MA) and RStudio (version 1.3.959; R version 4.0.2). Values reported are mean ± SEM unless otherwise stated.

## Supplementary Material

Supplementary data are available at *Genome Biology and Evolution* online.

## Supplementary Material

evac009_Supplementary_Data
