# Supplementary material for: Evolution of Transient Receptor Potential (TRP) Ion Channels in Antarctic Fishes (Cryonotothenioidea) and Identification of Putative Thermosensors
Source: Genome Biol Evol. 2022 Feb 2;14(2):evac009. doi: 10.1093/gbe/evac009 (PMC8857925; doi:10.1093/gbe/evac009)
Supplement: evac009_Supplementary_Data [file Supplemental figures and legends.pdf › Supplemental figures and legends.pdf]

Supplemental table and figure legends

Figure S1: complete maximum likelihood TRP channel protein tree, with labels. See Table S1 for data sources and species abbreviations and Table S2 for reference sequence and notothenioid accessions. Support values are from 200 bootstrap replicates. Scale bar indicates rate of amino acid substitutions per site.

Figure S2: *TRPM1* gene tree. *TRPM1a* and *TRPM1b* resolve as two distinct clades. Within the *TRPM1a* clade, *Chaenocephalus aceratus* (cace) and *Cottoperca gobio* (cgob) duplications cluster independently. Cryonotothenioid branches and nodes are blue. See Table S1 for species abbreviations. See genome mining tag of data repository for alignments.

Figure S3: *TRPV1* gene tree. *TRPV1a* and *TRPV1b* resolve as distinct clades with sequences from other teleosts. Cryonotothenioid branches and nodes are blue. See Table S1 for species abbreviations. See genome mining tag of data repository for alignments.

Figure S4: Verification of dissection and transcriptome data. (A) Correspondence analysis plotting the *Harpagifer* putative trigeminal ganglion dissection samples (black squares) and using ortholog expressed in cranial nerves of zebrafish (*Danio rerio*) for reference (red circles). (B) Library sizes for all samples from all tissues dissected from *Harpagifer antarcticus*. (C) Principle components analysis (PCA) comparing expression in all the *Harpagifer* samples. (D) Heat map plotting 1-Pearson correlation and cluster analysis for all samples. (E) PCA for all tissues dissected from *Astatotilapia burtoni*. (F) Heat map of 1-Pearson correlation for *Astatotilapia* samples.

Figure S5: All TRP channels that were significantly differentially expressed in *Harpagifer antarcticus* whole brain and trigeminal ganglion (TG) samples. Genes with significantly higher expression in the whole brain relative to the trigeminal ganglion shown in the top row, with adjusted p-values indicated. Counts are depth normalized and $\log_2$ transformed. Bottom row shows heat maps of differential gene expression (DGE) for the significant (adjusted $p<0.05$) TRP channel genes, unscaled on the right and scaled by row z-score ((gene expression for sample-mean expression for all samples)/standard deviation) on the left.

Figure S6: All TRP channel expression counts for *Astatotilapia burtoni* trigeminal ganglion TagSeq samples as mapped to the *A burtoni* genome.

Figure S7: Alignments of sites in TRPA1b, TRPV1a, TRPM4, and TRPM7 under significant diversifying selection in cryonotothenioids, according to MEME. Significant sites are indicated by asterisks and sites are numbered according to the human channel (TRPA1b, TRPV1a, TRPM4) or the *Oryzias latipes* channel (TRPM7). One site on either side of the significant site is shown for context. Reference sequences are in regular font, notothenioid species are bolded, cryonotothenioids are in blue. Accessions can be found in table S2.

Table S1: Existing sequence data sources and species abbreviations used in this study.

Table S2: Accessions for reference TRP channel sequences used to mine notothenioid transcriptomes and genomes and accessions for notothenioid sequences from transcriptome mining. See Table S1 for abbreviations. Note that “+” indicates that the sequences were sequential and non-overlapping so they were collated in the alignment. Sources for each transcriptome can be found in Table S1.

Table S3: Notes on genome mining analysis for all notothenioid species. Queries used for command line blast and results indicated. See Table S1 for genome sources and species abbreviations and Table 3 for gene counts.

Table S4: results from RepeatMasker for all species. Input data were scaffolds or chromosomes on which *TRPA1b* paralogs were found.

Table S5: Results from MEME analysis for all sites from all channels tested. Significant sites we noted with asterisks when the significant signal occurred within the cryonotothenioid clade.

References for supplemental material

Ahn, D.H., Shin, S.C., Kim, B.M., Kang, S., Kim, J.H., Ahn, I., Park, J., and Park, H. (2017). Draft genome of the Antarctic dragonfish Parachaenichthys charcoti. GigaScience. 6: 1-6.

Bilyk, K.T., Largas-Chacoff, L., and Cheng, C.H.C. (2018). Evolution in chronic cold: varied loss of cellular response to heat in Antarctic notothenioid fish. BMC Evolutionary Biology. 18: 143.

Shin, S.C., Ahn, D.H., Kim, S.J., Pyo, C.W., Lee, H., Kim, M.K., Lee, J., Lee, J.E., Detrich, H.W., Postlethwait, J.H., Edwards, D., Lee, S.G., Lee, J.H., and Park, H. (2014). The genome sequence of the Antarctic bullhead notothen reveals evolutionary adaptations to a cold environment. Genome Biology. 15: 468.

Figure S1

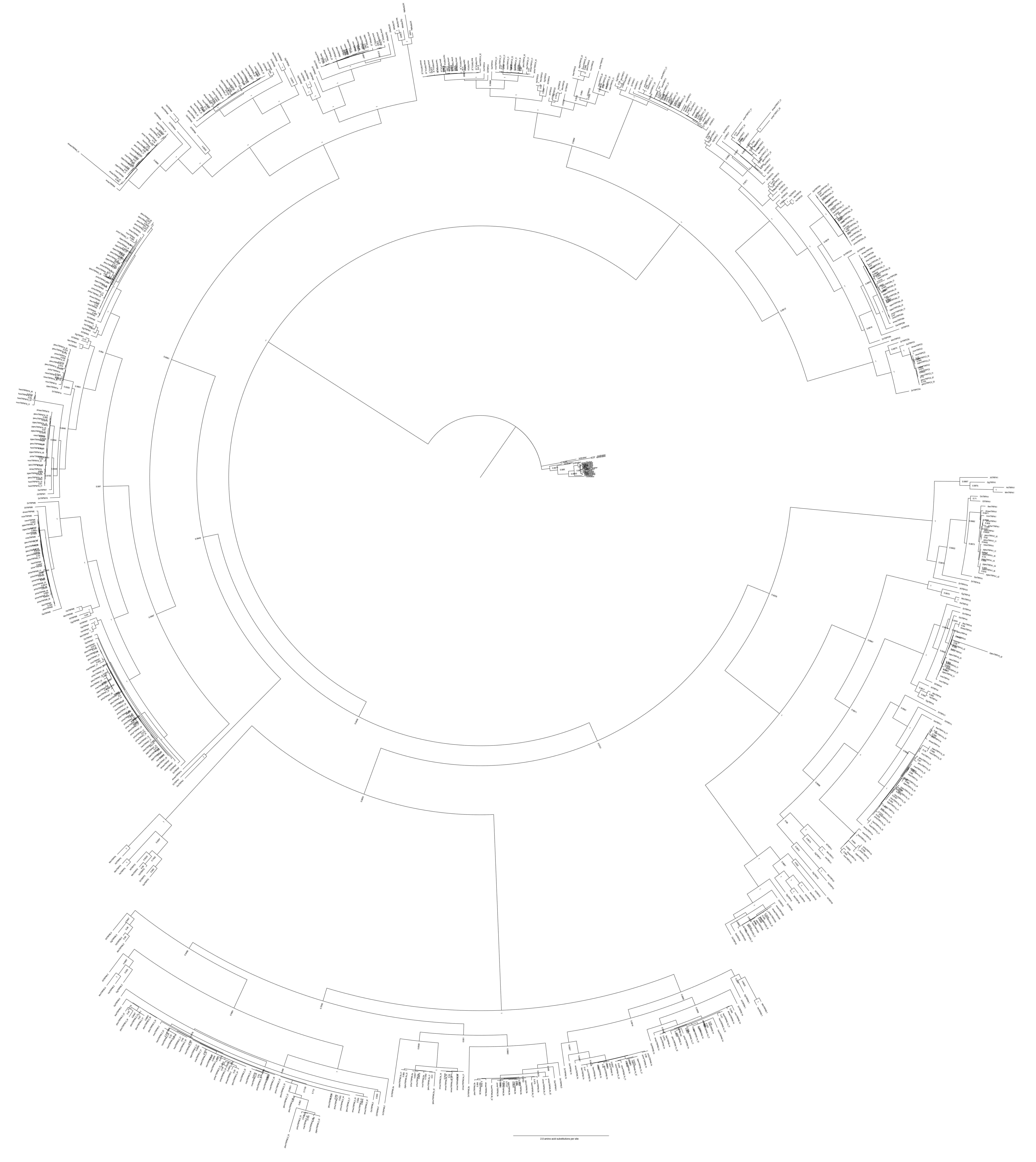

Figure S2

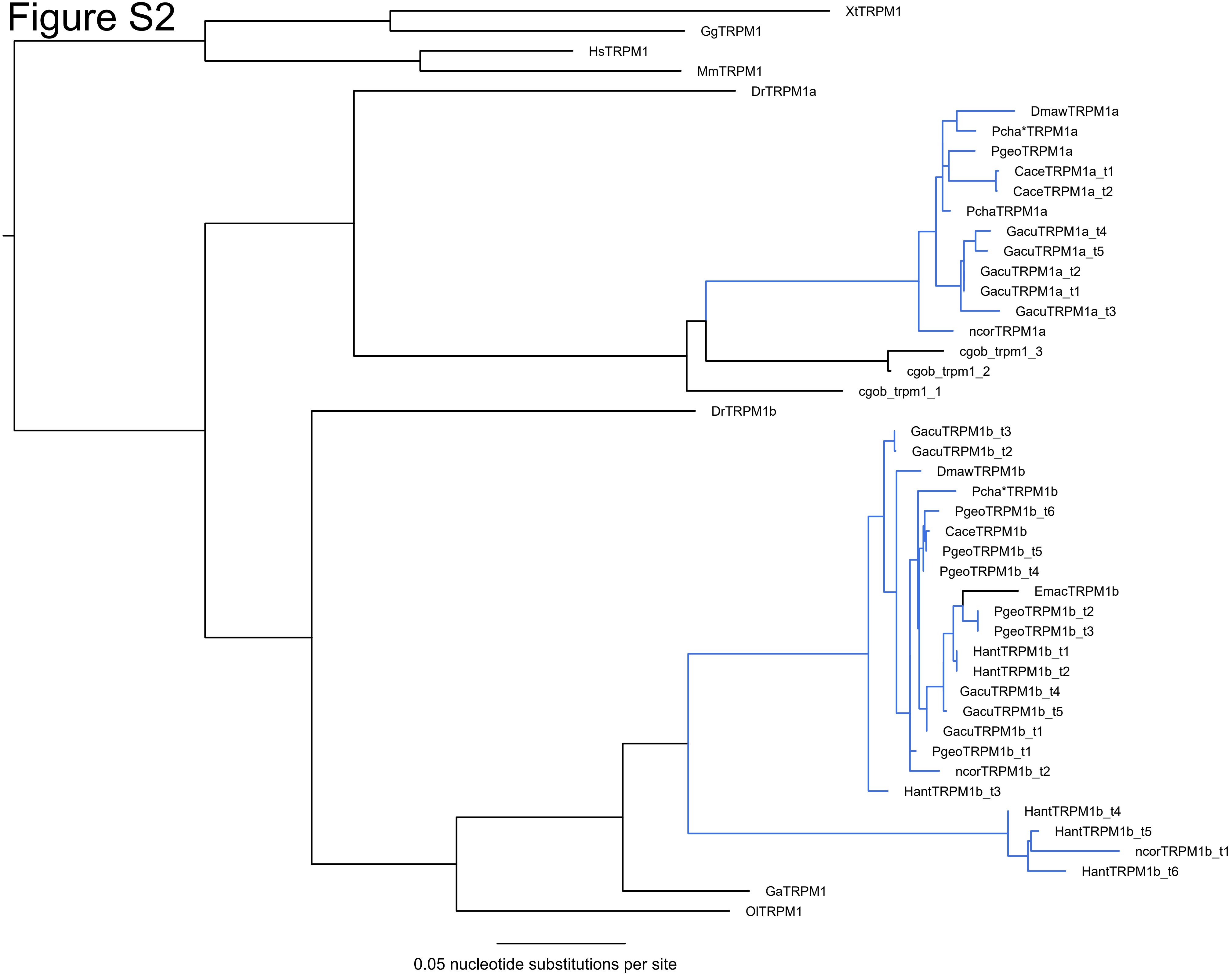
XtTRPM1
GgTRPM1
HsTRPM1
MmTRPM1
DrTRPM1a
DmawTRPM1a
Pcha*TRPM1a
PgeoTRPM1a
CaceTRPM1a_t1
CaceTRPM1a_t2
PchaTRPM1a
GacuTRPM1a_t4
GacuTRPM1a_t5
GacuTRPM1a_t2
GacuTRPM1a_t1
GacuTRPM1a_t3
ncorTRPM1a
cgob_trpm1_3
cgob_trpm1_2
cgob_trpm1_1
DrTRPM1b
GacuTRPM1b_t3
GacuTRPM1b_t2
DmawTRPM1b
Pcha*TRPM1b
PgeoTRPM1b_t6
CaceTRPM1b
PgeoTRPM1b_t5
PgeoTRPM1b_t4
EmacTRPM1b
PgeoTRPM1b_t2
PgeoTRPM1b_t3
HantTRPM1b_t1
HantTRPM1b_t2
GacuTRPM1b_t4
GacuTRPM1b_t5
GacuTRPM1b_t1
PgeoTRPM1b_t1
ncorTRPM1b_t2
HantTRPM1b_t3
HantTRPM1b_t4
HantTRPM1b_t5
ncorTRPM1b_t1
HantTRPM1b_t6
GaTRPM1
OlTRPM1
0.05 nucleotide substitutions per site

Figure S3

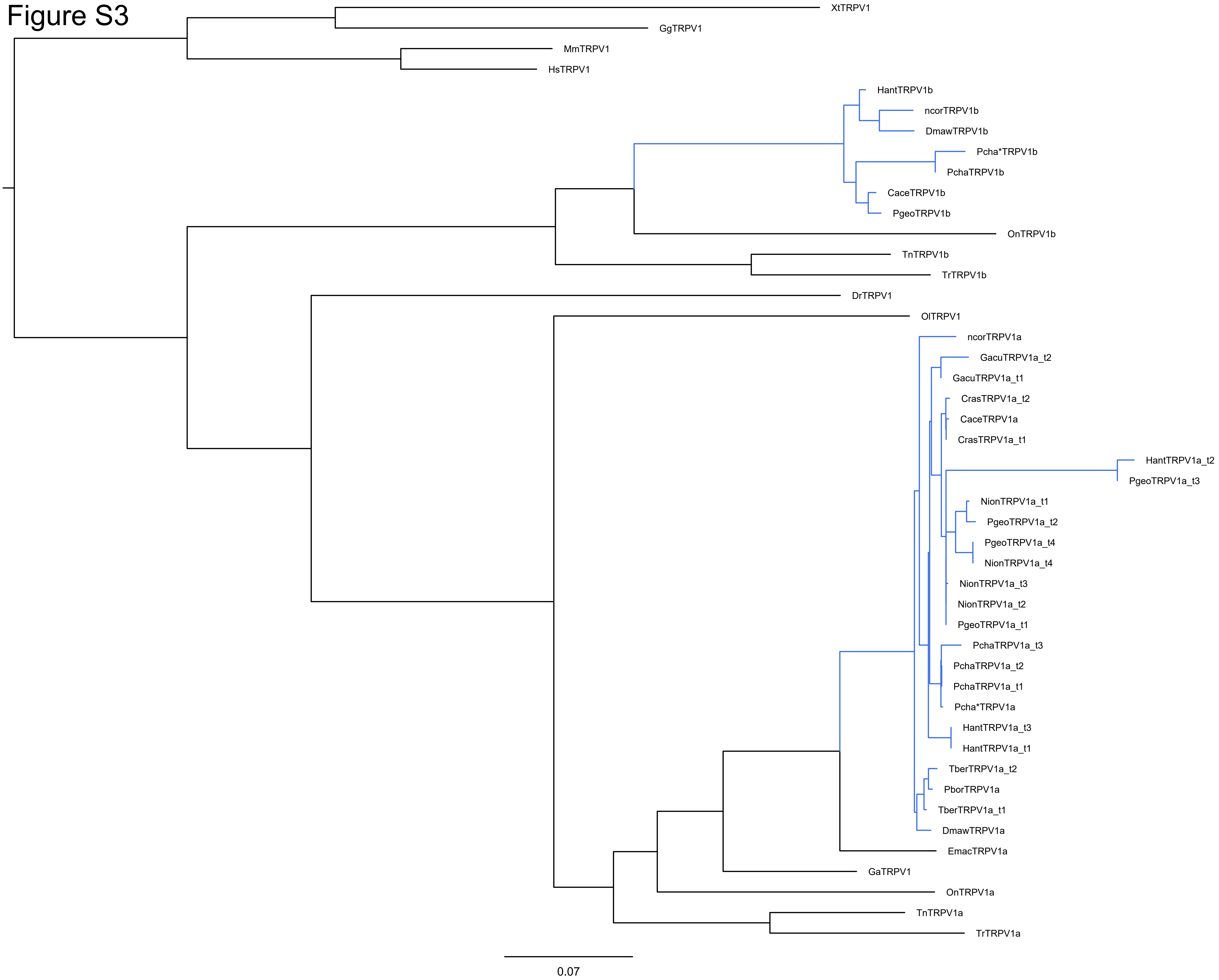
XtTRPV1
GgTRPV1
MmTRPV1
HsTRPV1
HantTRPV1b
ncorTRPV1b
DmawTRPV1b
Pcha*TRPV1b
PchaTRPV1b
CaceTRPV1b
PgeoTRPV1b
OnTRPV1b
TnTRPV1b
TrTRPV1b
DrTRPV1
OlTRPV1
ncorTRPV1a
GacuTRPV1a_t2
GacuTRPV1a_t1
CrasTRPV1a_t2
CaceTRPV1a
CrasTRPV1a_t1
HantTRPV1a_t2
PgeoTRPV1a_t3
NionTRPV1a_t1
PgeoTRPV1a_t2
PgeoTRPV1a_t4
NionTRPV1a_t4
NionTRPV1a_t3
NionTRPV1a_t2
PgeoTRPV1a_t1
PchaTRPV1a_t3
PchaTRPV1a_t2
PchaTRPV1a_t1
Pcha*TRPV1a
HantTRPV1a_t3
HantTRPV1a_t1
TberTRPV1a_t2
PborTRPV1a
TberTRPV1a_t1
DmawTRPV1a
EmacTRPV1a
GaTRPV1
OnTRPV1a
TnTRPV1a
TrTRPV1a
0.07

Figure S4

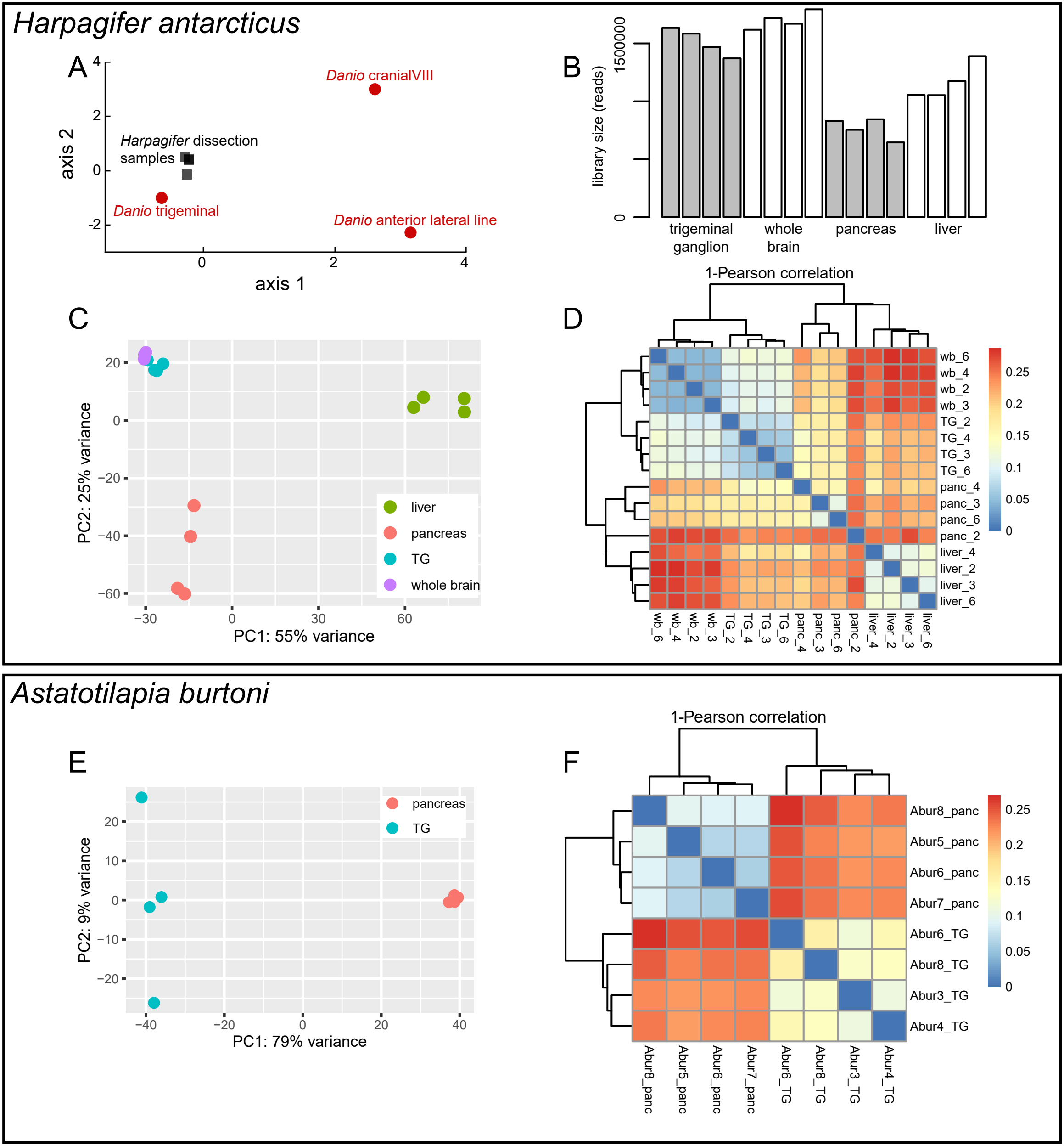

Harpagifer antarcticus
A
Danio cranialVIII
Harpagifer dissection samples
Danio trigeminal
Danio anterior lateral line
axis 1
axis 2
B
library size (reads)
1500000
0
trigeminal ganglion
whole brain
pancreas
liver
C
PC1: 55% variance
PC2: 25% variance
liver
pancreas
TG
whole brain
D
1-Pearson correlation
wb_6
wb_4
wb_2
wb_3
TG_2
TG_4
TG_3
TG_6
panc_4
panc_3
panc_6
panc_2
liver_4
liver_2
liver_3
liver_6
Astatotilapia burtoni
E
PC1: 79% variance
PC2: 9% variance
pancreas
TG
F
1-Pearson correlation
Abur8_panc
Abur5_panc
Abur6_panc
Abur7_panc
Abur6_TG
Abur8_TG
Abur3_TG
Abur4_TG

Figure S5

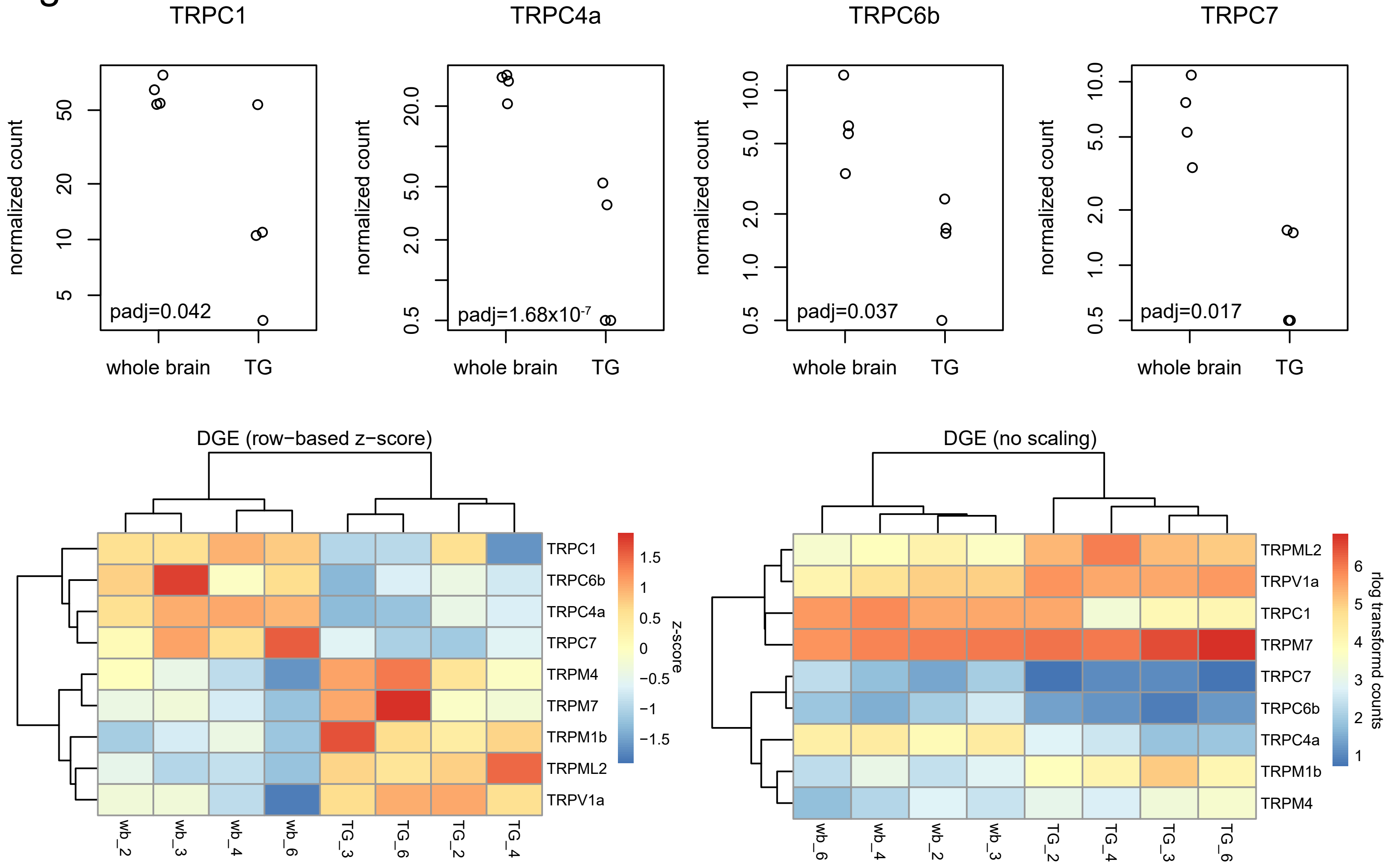

TRPC1
TRPC4a
TRPC6b
TRPC7
normalized count
padj=0.042
padj=1.68x10-7
padj=0.037
padj=0.017
whole brain
TG
DGE (row−based z−score)
DGE (no scaling)
TRPC1
TRPC6b
TRPC4a
TRPC7
TRPM4
TRPM7
TRPM1b
TRPML2
TRPV1a
z-score
wb_2
wb_3
wb_4
wb_6
TG_3
TG_6
TG_2
TG_4
wb_6
wb_4
wb_2
wb_3
TG_2
TG_4
TG_3
TG_6
rlog transformd counts

Figure S6

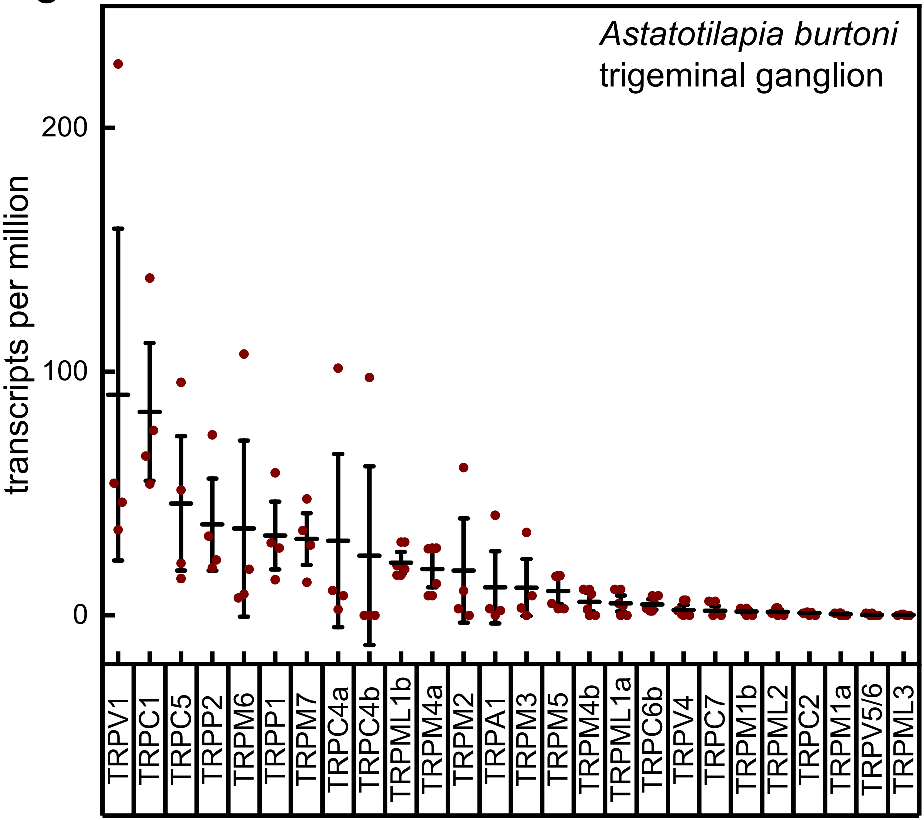

Astatotilapia burtoni
trigeminal ganglion
transcripts per million
200
100
0
TRPV1
TRPC1
TRPC5
TRPP2
TRPM6
TRPP1
TRPM7
TRPC4a
TRPC4b
TRPML1b
TRPM4a
TRPM2
TRPA1
TRPM3
TRPM5
TRPM4b
TRPML1a
TRPC6b
TRPV4
TRPC7
TRPM1b
TRPML2
TRPC2
TRPM1a
TRPV5/6
TRPML3
